# Identification of intestinal microbiome associated with lymph-vascular invasion in colorectal cancer patients and predictive label construction

**DOI:** 10.3389/fcimb.2023.1098310

**Published:** 2023-05-12

**Authors:** Chuanbin Chen, Kang Chen, Zigui Huang, Xiaoliang Huang, Zhen Wang, Fuhai He, Mingjian Qin, Chenyan Long, Binzhe Tang, Xianwei Mo, Jungang Liu, Weizhong Tang

**Affiliations:** Division of Colorectal & Anal Surgery, Department of Gastrointestinal Surgery, Guangxi Medical University Cancer Hospital, Nanning, China

**Keywords:** colorectal cancer, lymph-vascular invasion, 16S rRNA, intestinal microbiome, machine learning

## Abstract

**Objective:**

To identify differences between the composition, abundance, and biological function of the intestinal microbiome of patients with and without lymph-vascular invasion (LVI) colorectal cancer (CRC) and to construct predictive labels to support accurate assessment of LVI in CRC.

**Method:**

134 CRC patients were included, which were divided into two groups according to the presence or absence of LVI, and their intestinal microbiomes were sequenced by 16SrRNA and analyzed for differences. The transcriptome sequencing data of 9 CRC patients were transformed into immune cells abundance matrix by CIBERSORT algorithm, and the correlation among LVI-associated differential intestinal microbiomes, immune cells, immune-related genes and LVI-associated differential GO items and KEGG pathways were analyzed. A random forest (RF) and eXtreme Gradient Boosting (XGB) model were constructed to predict the LVI of CRC patients based on the differential microbiome.

**Result:**

There was no significant difference in α-diversity and β-diversity of intestinal microbiome between CRC patients with and without LVI (P > 0.05). Linear discriminant analysis Effect Size (LEfSe) analysis showed 34 intestinal microbiomes enriched in CRC patients of the LVI group and 5 intestinal microbiomes were significantly enriched in CRC patients of the non-lymph-vascular invasion (NLVI) group. The RF and XGB prediction models constructed with the top 15% of the LVI-associated differential intestinal microbiomes ranked by feature significance had good efficacy.

**Conclusions:**

There are 39 intestinal flora with significantly different species abundance between the LVI and NLVI groups. *g:Alistipes.s:Alistipes_indistinctus* is closely associated with colorectal cancer vascular invasion. LVI-associated differential intestinal flora may be involved in regulating the infiltration of immune cells in CRC and influencing the expression of immune-related genes. LVI-associated differential intestinal flora may influence the process of vascular invasion in CRC through a number of potential biological functions. RF prediction models and XGB prediction models constructed based on microbial markers of gut flora can be used to predict CRC-LVI conditions.

## 1 Introduction

Colorectal cancer (CRC) is a great threat to human health, with the third and second highest incidence and mortality rates, respectively, according to the 2020 Global Cancer Data estimates, and its incidence continues to increase in many countries (Sung et al., 2021). In recent years, the incidence of CRC has increased significantly in China, with 592,232 incidences and 309,114 deaths expected in 2022 (Xia et al., 2022). Despite breakthroughs in both surgical and medical treatments for CRC, there has been no significant improvement in patient cure rates or long-term survival (Kuipers et al., 2015).

The etiology of CRC is unknown, but genetic and environmental factors have emerged as well-recognized risk factors for the development of CRC (Kuipers et al., 2015). In recent years, the intestinal microbiome and their metabolites have been shown to influence the development and progression of CRC by mediating or modifying environmental factors or by being modified by environmental factors (Song et al., 2020). The normal human gut contains approximately 100 trillion commensal intestinal bacteria (Hill and Artis, 2010), expressing over 3.3 million genes (Belizário and Napolitano, 2015). The intestinal microbiome plays an important role in digestion and absorption, substance metabolism, immune regulation, and protection of the intestinal mucosa, and together they maintain intestinal homeostasis (Hill and Artis, 2010; Belizário and Napolitano, 2015; Okumura and Takeda, 2018). The intestinal microbiome in healthy populations is mainly composed of Bacteroidetes and Firmicutes (Mahowald et al., 2009), among which Bifidobacterium and Lactobacillus as probiotics can participate in the host’s gastrointestinal defense system (Servin, 2004), and Clostridium nucleatum or Bacteroides fragilis as harmful bacteria have been shown to be associated with the occurrence and development of CRC (Montalban-Arques and Scharl, 2019). Therefore, the composition ratio and relative abundance of the intestinal microbiome are crucial for maintaining the stability of the intestinal micro-ecosystem and human health. In recent years, due to the improvement in living standards, people have increased the intake of red meat and animal fat and reduced the intake of fiber in their diets and decreased the amount of exercise in their lifestyles. Since the composition of the microbiome is closely related to the host’s lifestyle, diet and genotype, these lifestyle changes, especially the diet, have altered the composition of the intestinal microbiome and caused an imbalance in the intestinal microbiome (Yang and Yu, 2018; Redondo-Useros et al., 2020). Intestinal microbiome imbalance refers to a change in the predominant microbiome of the original intestine, a decrease in the abundance of the beneficial microbiome, and an increase in the abundance of the pathogenic microbiome in the intestinal microecological environment, which will cause chronic inflammation and genetic alterations in colorectal epithelial cells through the release of virulence factors, leading to CRC (Wang and Li, 2022).

Currently, due to the lack of early symptoms of CRC (Tepus and Yau, 2020), most patients are diagnosed when CRC has progressed to the progressive stage (Yu YQ. et al., 2022), suggesting that the tumor has metastasized, which is a major cause of cancer-related death (Vatandoust et al., 2015). Although the relationship between intestinal microbiome imbalance and CRC occurrence has been extensively studied, the specific relationship between various bacteria and CRC metastasis has still not been elucidated. Metastasis is a complex multigene, multistep process consisting of close communication between tumor cells and the tumor microenvironment, including immune cells, inflammatory cells, and stromal cells. A study showed that lipopolysaccharide increases VEGF-C secretion through the TLR4- NF-κ b/JNK signaling pathway and promotes cell motility and lymphangiogenesis (Zhu et al., 2016). The presence of lipopolysaccharide in the outer membrane of Gram-negative bacteria suggests that the intestinal microbiome plays an important role in the lymphatic metastasis of CRC. The most common site of hematogenous metastasis in CRC is the liver (Pretzsch et al., 2019). About 25% of CRC patients accompany with liver metastases, which causes more than 90% of deaths in CRC patients (Li et al., 2019). Bullman, S found that Clostridium nucleolytic and its commensal microorganisms (Mycobacterium, Puccinia) colonize distal liver metastases and that the microorganisms present in liver metastases colonized by Clostridium nucleolytic are similar to those associated with Clostridium nucleolytic in primary CRC (Bullman et al., 2017). Another animal study showed that Desulfovibrio can create a microenvironment conducive to CRC liver metastasis by inducing colonic barrier dysfunction, colonic and hepatic inflammation (Yu Y. et al., 2022).

Invasion of lymphatic and blood vessels by tumor cells plays a crucial role in the process of metastasis (van Zijl et al., 2011). Lymph-vascular invasion (LVI) is defined as a pathological manifestation of the presence of cancer thrombi in lymphatic or vascular channels (Li et al., 2015), and LVI is an early pathological marker of tumor progression to metastasis (Schneider and Langner, 2014). It has been well documented that LVI of tumor tissue (including both lymphatic vessel invasion and vascular invasion) is an independent predictor of poor prognosis in CRC and that CRC patients with LVI have a poorer prognosis compared to non-lymph-vascular invasion (NLVI) CRC patients. Therefore, elucidating the oncologic impact and prognostic significance of LVI is of great significance for CRC patients.

There are no studies showing the relationship between the gut microbiome and LVI. The aim of this study was to investigate the composition, abundance, and biological functions of each intestinal microbiome in LVI-CRC patients and NLVI-CRC patients, and to incorporate the characteristic microbiome to construct a machine learning classification model to analyze the role of gut microbiota in the development of LVI in CRC, to identify novel noninvasive biomarkers of CRC-LVI, and to construct a diagnostic model to assess CRC-LVI conditions to provide new strategies for early screening of CRC metastasis.

## 2 Research methods

### 2.1 Subject information and sample collection

The study protocol was approved by the Medical Ethics Committee of the Affiliated Cancer Hospital of Guangxi Medical University, and all participants were notified prior to sample collection. All study subjects signed an informed consent form before any procedures were performed. The investigators had completed the collection of pre-treatment stool samples from 236 colorectal cancer patients between 2021.01.01 and 2021.12.31 after inclusion and exclusion screening, and finally received 198 stool samples of acceptable quality for 16S rRNA sequencing. Among the above subjects who underwent surgical treatment at the Cancer Hospital of Guangxi Medical University, fresh tissue specimens removed by surgery were collected and placed in liquid nitrogen for preservation. Among them, there were 134 colorectal cancer patients with LVI information. In addition, we performed transcriptome sequencing on 17 colorectal cancer tumor tissue samples, among which 15 samples had LVI information, and 9 samples had 16S rRNA sequencing data from stool samples.

Subjects were included according to the following criteria: 1. Patients who had undergone surgical treatment with clear pathological staging (according to ACJJCRC staging guidelines), or patients with colorectal adenocarcinoma confirmed by colonoscopic pathological biopsy; 2. No combination or no previous other malignancies; 3. Exclusion of other intestinal diseases and no acute comorbidities such as complete intestinal obstruction, intestinal perforation, or pelvic abscess; 4. Before fecal sample collection, all patients had not received any anti-tumor treatment, such as surgery, chemotherapy, radiotherapy, immunotherapy and traditional Chinese medicine; 5. No antibiotics and intestinal microecological agents were used in the past 1 month; 6. No impairment of consciousness or other cognitive dysfunction.

Stool specimen collection: Stool specimens were collected on the first day after the patients were admitted to the hospital. Patients were instructed to use a sterile stool collection tube to retain the middle part of the stool specimen and avoid contamination of urine, then the stool specimen was placed in a sterile ice box, dispensed in 2 mL EP tubes, 200 mg/tube, and placed in -80 °C refrigerator for freezing and storage.

Tissue sample collection: Fresh tissue specimens were collected from surgically excised tumors and paracancerous tissues of soybean size, and the time from isolation to placement in liquid nitrogen for preservation was controlled to be less than 30 minutes.

### 2.2 16S rRNA sequencing

Using the MOBIO PowerSoil^®^ DNA Isola-tion Kit, DNA was extracted from 200 mg of feces in Tris-EDTA buffer according to the product instructions. After DNA extraction, the samples are tested for DNA quality, and samples of acceptable quality are allowed to proceed to the next experiment. The V3 and V4 regions of the 16S rRNA gene were targeted and captured with primers 341F: (5′-CCTACGGGNGGCWGCAG-3′) and 805R (5′-GACTACHVGGGTATCTAATCC-3), and the targeted capture products were amplified by PCR. After PCR amplification, the PCR products of each sample were first examined using 2% agarose gel electrophoresis with a target band size of approximately 300 ~ 350 bp for the target capture products. The PCR products were then quantified using the Quant-iT PicoGreen dsDNA Assay Kit kit, and all samples were combined at equimolar concentrations according to the sequencing requirements based on the quantitative results of each sample. Next, the mixed libraries were quantified using the KAPA Library Quantification Kit KK4824. Finally, the libraries were sequenced on an Illumina PE250 instrument using 2×250 bp chemistry after passing the library assay.

### 2.3 Transcriptome sequencing

Total RNA was extracted from 17 colorectal cancer tumor samples using Trizol^®^ Total RNA Extraction Kit, and electrophoresis was used to detect the integrity of RNA, and the purity of RNA was measured by micro UV spectrophotometer. Remove rRNA and construct cDNA libraries by referring to the instructions of the RNA-seq Sample Preparation Kit (VAHTS™ Stranded mRNA-seq Library Prep Kit for Illumina^®^). Transcriptome library sequencing was done by Illumina NovaSeq 6000. The sequenced raw data were quality assessed by FastQC, and the sample valid data were first compared to the reference genome using HISAT2 (version: hg38). Gene expression was assessed using StringTie and known gene models, and the TPM (Transcripts Per Million) calculated for each gene was used as the expression abundance of that gene.

### 2.4 Analysis of tumor immune infiltration

The tumor immune infiltration analysis was performed by calling CIBERSORT R script v1.03. The CIBERSORT algorithm uses the microarray data to construct a feature matrix that translates the TPM matrix into a relative content matrix of 22 immune cells (including immune cells of different cell types and functional states) (Newman et al., 2015).

### 2.5 Functional enrichment analysis of LVI-related transcriptome sequencing

The single-sample gene set enrichment analysis (ssGSEA) algorithm is a rank-based approach that calculates a score for the absolute enrichment of a specific gene set for each sample (Hänzelmann et al., 2013). We downloaded the required gmt format gene set files (c2.cp.kegg.v2022.1.Hs.symbols.gmt, c5.go.v2022.1.Hs.symbols. gmt), using the ssgsea algorithm to calculate the gene set scoring matrix for each sample by the GSVA package v1.46.0. Next, the NLVI group was used as a control group, and the limma algorithm in TCGAbiolinks package v2.25.3 was used to analyze the differential GO items and KEGG pathways between groups. GO analysis includes three levels: Biological Process (BP), Molecular Function (MF) and Cellular Component (CC). The significance thresholds for differentially expressed genes were: p value< 0.05 and |log2FC| >0. log2FC>0 indicates genes upregulated in the LVI group and log2FC<0 indicates genes upregulated in the NLVI group, and the screening process was presented by volcano plot, depicted based on ggplot2 package v3.4.0.

### 2.6 Machine learning model construction and identification of gut microbial markers

We used Random Forest (RF) and XGBoosting (eXtreme Gradient Boosting, XGB) models for gut microbial marker identification to predict LVI in CRC patients, respectively.

The RF model and XGB model are widely used machine learning methods with promising prediction results (AlThuwaynee et al., 2021; Hong et al., 2022; Yu JR. et al., 2022). RF model is an integrated machine learning approach by building a large number of decision trees (Breiman, 2001). The method is evaluated by combining multiple decision trees in an integrated manner, and the generalization error converges as the number of trees increases, so the algorithm does not suffer from overfitting problems (Breiman, 2001). Compared with other models, the RF model can handle a large number of interactions between different independent variables, so the algorithm does not have the problem of multicollinearity (Breiman, 2001; Hajipour et al., 2020). To sum up, RF model usually has higher accuracy than other machine learning methods (Breiman, 2001; Hajipour et al., 2020; Pluth and Brose, 2022). XGB is a gradient-enhanced integrated learning model that focuses on training multiple weak classifiers and assembling them into a stronger classifier with the goal of minimizing the loss function and increasing the weight of misclassifications by computing negative gradients to improve training for the next iteration (Krishnapuram et al., 2016). Compared with the traditional gradient boosting decision tree (GBDT), XGB adds a regularization method, which makes the loss function smoother, reduces the complexity of the model, and avoids model overfitting (Chang et al., 2022). In addition, XGB uses an approximation algorithm to find the optimal solution for segmentation, and optimizes gradient enhancement to improve efficiency and scalability (Yu JR. et al., 2022).

We applied Python and SciKit Learn 0.18 (https://scikit-learn.org/stable/) platforms to install and load the installation packages used to build and evaluate machine learning models with default parameters for model construction. The gut flora data of 134 CRC patients with LVI information were used as samples, randomly divided into training and test sets by 7:3, and the species of gut flora were used as features of the data, and the RF and XBG models were constructed and predicted based on the top 15% species of feature importance among LVI-related differential gut bacteria, respectively, using the subject operating curve (ROC) and under curve area under the curve (AUC) to assess the accuracy of the machine learning models on the training and validation sets.

### 2.7 Analytical methods for 16S rRNA sequencing

The following operations were performed in R software v3.5.1. All P values are two-tailed and are treated as statistically significant at P < 0.05. After obtaining the FASTQ raw sequencing data for each sample, the raw sequencing data were quality filtered using Quantitative In-sights Into Microbial Ecology version 2 (QIIME2), species annotation was performed based on the Greengene database v13.8, and the phyloseq package was used to v1.26.1 for gut flora ASV/OTU extraction. First, the species diversity of the intestinal flora of each sample was measured as alpha diversity, with the Chao1 and ACE indices describing the flora richness (Community richness), and the Shannon and Simpson indices describing the species diversity and homogeneity of the flora. The variability in species composition of the gut flora of each sample in the same ecology was measured by beta diversity, and ADONIS analysis and ANOSIM analysis in beta diversity analysis were performed by vegan package v2.5.6. The Partial Least squares-discriminant Analysis (PLS-DA) of fecal intestinal flora was performed by the package mixOmics v6.6.2. LEfSe analysis supports high-dimensional taxonomic comparisons (Zhang et al., 2013) and can be used to screen for species that are most likely to explain differences between groups. Next, LEfSe analysis was performed using Lefse software v1.0.0, and the results of the LEfSe analysis were evaluated using linear discriminant analysis (LDA) to assess the effect values for each species that differed significantly (i.e., LDA score, which by default undergoes log transformation with a base of 10, the larger the absolute value the easier it is to distinguish between groups). Species with significant differences in abundance between groups were obtained using |LDA|>2 and P<0.05 as the difference screening threshold, and the results were presented in a bar graph. Finally, PICRUSt2 software 2.3.0 was used to predict the KEGG pathway enriched between groups for the sequenced samples. The analysis of variability of α and β diversity indices and KEGG pathways between groups were performed using the nonparametric Mann-Whitney U rank sum test, both using the vegan package v2.5.6, and the visualization of histograms was done by the ggplot2 package v3.4.0.

### 2.8 Statistical methods

All the following operations were analyzed by R software v4.2.2. All P values are two-tailed and are treated as statistically significant at P < 0.05. Continuous data analysis of clinical data was performed by t-test and quantitative data analysis was performed by Pearson chi-square test (**Table 1**), which was calculated by SPSS software v23.0. Correlations between intestinal flora and immune cell abundance and immune-related genes were calculated using Pearson correlation, correlations between different subgroups of dominant flora, and correlations between intestinal flora and KEGG pathway were calculated using Spearman correlation, all by Hmisc package v4.7-1. Correlations between gut flora and BP items and MF items were calculated using Spearman correlations through the ggcorrplot package v0.1.4. and the correlation matrix was visualized using ggcorrplot package v0.1.4, Igraph package v1.3.5 and Cystoscope software Version 3.7.2.

**Table 1:** Demographic and clinical characteristics of CRC patients stratified by LVI condition.

		CRC patients with LVI (n=47)	CRC patients with NLVI (n=87)	P value	Test
Age (years, mean (SD))		57.96 ± 11.46	57.85 ± 10.75	0.957	T-Test
Age (%)	**≥60**	21 (44.70)	37 (42.50)	0.810	Pearson Chi-square
	**<60**	26 (55.30)	50 (57.50)		
Gender (%)	male	28 (59.60)	55 (63.20)	0.678	Pearson Chi-square
	female	19 (40.40)	32 (36.80)		
TNM stage (%)	**early (0~2)**	15 (31.90)	44 (51.20)	0.033*	Pearson Chi-square
	**advanced (3~4)**	32 (68.10)	42 (48.80)		
perineural invasion (%)	**YES**	41 (89.10)	32 (37.20)	<0.001***	Pearson Chi-square
	**NO**	5 (10.90)	54 (62.80)		

The “*” in the upper right corner of the P value value indicates the P value: none* for P value ≥ 0.05, * for 0.01 ≤ P < 0.05, ** for 0.001 ≤ P < 0.01, *** for 0.0001 ≤ P < 0.001.

## 3 Result

### 3.1 Demographic and clinical characteristics of CRC patients stratified by LVI condition

A total of 134 CRC patients with information on vascular invasion were included in this study. Among them, 47 patients with CRC with vascular invasion and 87 patients with CRC without vascular invasion were included. As shown in **Table 1**, there were no statistically significant differences in age and sex between CRC patients with and without vascular invasion, indicating that the baseline data were balanced and comparable. The proportion of CRC patients with vascular invasion with TNM stage III-IV (P=0.033) and with nerve invasion (P<0.001) was significantly higher than that of CRC patients without vascular invasion, suggesting that LVI is associated with the progression of CRC.

### 3.2 Comparison of microbiome diversity between CRC patients with LVI and NLVI

**Figure 1:**
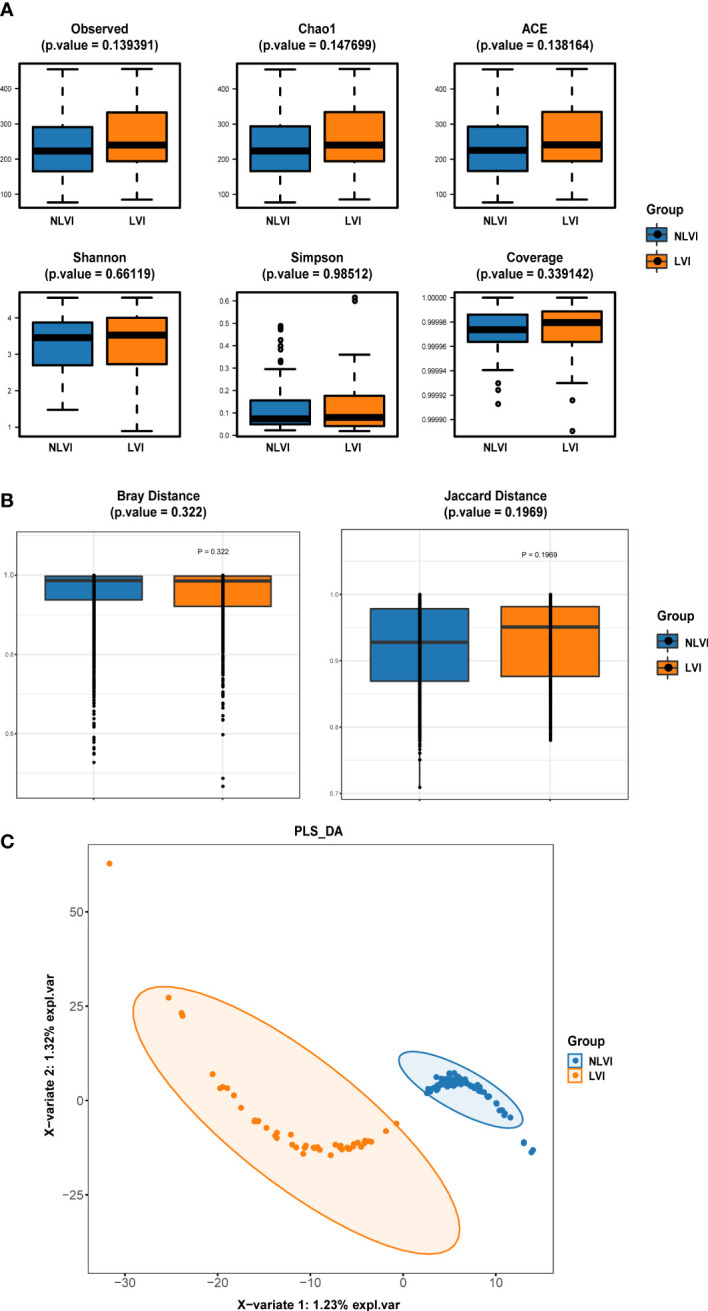
Diversity index of intestinal flora in the LVI group compared with the NLVI group of CRC patients **(A)** Comparison of alpha diversity index of intestinal flora between LVI group and NLVI group of CRC patient group. **(B)** Comparison of β diversity index of intestinal flora in LVI group and NLVI group of CRC patient group. The horizontal coordinates indicate the group, the vertical coordinates indicate the value of community diversity index of the samples in this group, and the color indicates the group. **(C)** PLS-DA analysis of intestinal flora in the LVI group versus the NLVI group of CRC patients. The dots represent each gut flora sample, the color indicates the group, the scale of horizontal and vertical axes indicates the relative distance of each sample, and X-variate 1 and X-variate 2 represent the factors affecting the shift of gut flora composition in CRC patients in LVI and NLVI groups, respectively.

First, we investigated whether there was a difference in the diversity of the flora between the LVI and NLVI groups of patients, which was mainly measured by the alpha diversity and versus beta density indices. **Figure 1A** compares the 6 alpha diversity indices of the intestinal flora samples of CRC patients in the LVI and NLVI groups, but none of the differences were statistically significant (P > 0.05). The beta diversity of the CRC patient groups in the LVI and NLVI groups is shown in **Figure 1B**, and there were no statistical differences in the Bray (P=0.322) and Jaccard’s test indices (P=0.197) between the gut microbial composition of the two groups (see **Supplementary Tables 1**, **2**). PLS-DA analysis suggested that the LVI and NLVI groups could be distinguished into two distinct groups (**Figure 1C**). The above results showed that there were no significant differences in species diversity and community composition of fecal microbial communities between the LVI and NLVI groups, but there were still clear group differences in the composition of the intestinal flora between the LVI and NLVI groups.

### 3.3 Identification of intestinal microbiome associated with LVI condition

**Figure 2:**
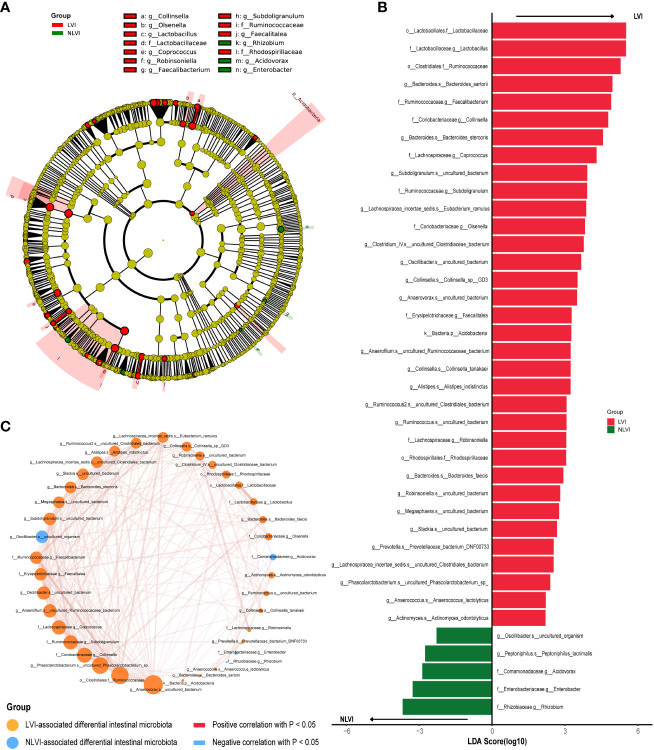
Differential intestinal flora analysis of CRC patients in the LVI and NLVI groups **(A)** Evolutionary relationship diagram for LEfSe analysis. Node size represents species abundance size, which is proportional to species abundance size. Node color represents grouping, yellow nodes in the branches indicate species that do not differ significantly in abundance between groups; red nodes indicate species with significantly higher abundance in the LVI group, and green nodes indicate species with significantly higher abundance in the NLVI group. The nodes in each layer indicate the phylum/class/order/family/genus/species from inside-out, and the annotations of species markers in each layer indicate the phylum/class/order/family/genus/species from outside-in. **(B)** LDA bar graph based on 16S rRNA gene sequencing. The color of the bar graph represents the group, the horizontal coordinate is the LDA score (after log10 processing), the vertical coordinate indicates the differential species with significantly higher abundance in the group, and the length of the bar graph represents the size of the LDA score value. **(C)** Network diagram of LVI-related differential gut flora correlation. Each node represents each species, node color represents grouping, node size represents the number of edges connected to the node, the larger the node, the more the number of edges connected to the node, the connecting line represents the existence of significant correlation between two nodes, Spearman correlation coefficient values below 0 (negative correlation) represent the blue line, Spearman correlation coefficient values greater than 0 (positive correlation) represent the red line The thicker the line, the larger the Spearman correlation coefficient between the two nodes.

To search for potential LVI-associated intestinal flora biomarkers (species with significant differences in abundance between the LVI and NLVI groups), we performed LEfSe analysis of fecal microorganisms from CRC patients in the LVI and NLVI groups. Among them, we found statistically significant differences in the abundance of a total of 39 species. There were a total of 34 species in the LVI group with significantly higher abundance than the NLVI group, and a total of 5 species in the NLVI group with significantly higher abundance than the LVI group (P < 0.05, as in **Supplementary Table 3**; **Figures 2A, B**). The LDA bar chart in **Figure 2B** shows the LDA scores of the LEfSe analysis for each important colony (after taking log10 processing), with higher scores indicating the greater influence of the species. In addition, to explore the interaction between LVI-related differential flora, we depicted the correlation graph between the dominant flora of the LVI group and the dominant flora of the NLVI group (**Figure 2C**). Among them, *g_Anaerovorax.s_uncultured_bacterium,_o_Clostridiales.f_Ruminococcaceae, g_Phascolarctobacterium.s_uncultured_Phascolarctobacterium_sp., f_Coriobacteriaceae.g_Collinsella* and *f_Lachnospiraceae.g_Coprococcus* were the five most connected bacterias with other nodes. It indicates that these five groups have the closest correlation with other dominant bacteria. Moreover, there was a significant negative correlation between the dominant flora *f_Comamonadaceae.g_Acidovorax* and *f_Rhizobiaceae.g_Rhizobium* in the NLVI group and some of the dominant flora of the LVI group. The above results suggest that there may be a competitive relationship between these dominant bacterial groups and each other.

### 3.4 Function prediction of intestinal microbiome between CRC patients with LVI and NLVI

To investigate the biological pathways enriched by LVI-related intestinal flora genes, we used PICRUSt2 (Phylogenetic Investigation of Communities by Reconstruction of Unobserved States 2) software to predict the KEGG pathways between the LVI and NLVI groups of flora. A total of 173 KEGG pathways were identified, 47 of which were statistically significantly different (P<0.05). The LVI group had 33 KEGG pathways in significantly higher abundance than the NLVI group, and the three most significant pathways were Dioxin degradation (P=0.004), Phosphotransferase system (PTS) (P= 0.008) and Caprolactam degradation (P= 0.013). The NLVI group had 14 KEGG pathways in significantly higher abundance than the LVI group. Non-homologous end-joining (P=0.004), Glycerophospholipid metabolism (P=0.040) and Methane metabolism (P=0.044) were three of the most significant pathways (see **Supplementary Figure 1**, **Supplementary Table 4** for details, P < 0.05). The above results suggest that LVI-associated intestinal flora have differential metabolic functions.

### 3.5 Relationship between LVI-associated differential intestinal flora and tumor-infiltrating immune cells

**Figure 3:**
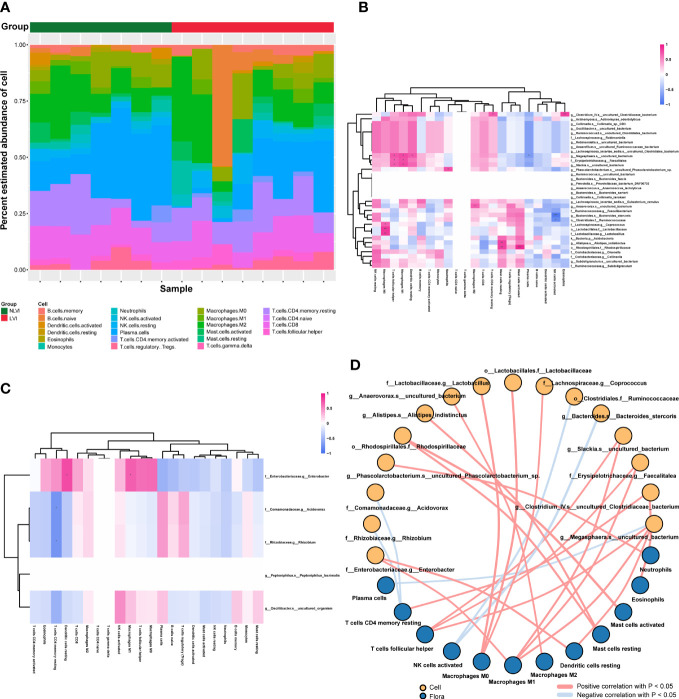
Correlation of LVI-related differential intestinal flora with tumor immune infiltrating cells **(A)** Bar graph of relative abundance of immune cells in CRC patients grouped by LVI status. Each bar is one sample, the vertical coordinate is the predicted relative abundance value of immune cells, the sum of the relative abundance of all immune cells in a single sample is 1, and each color in the graph corresponds to one immune cell. **(B)** Heat map of the correlation between the dominant flora and the abundance of immune cells in the LVI group. **(C)** Heat map of the correlation between the dominant flora and the abundance of immune cells in the NLVI group. The horizontal coordinates are immune cells, the vertical coordinates are bacteria, the red color in the graph represents positive correlation, the blue color represents negative correlation, the color depth represents the Pearson correlation coefficient size, the color from light to dark indicates the Pearson correlation coefficient value from small to large. The “*” in the graph indicates the size of P-value: no * for P-value ≥ 0.05, * for 0.01 ≤ P<0.05, ** for 0.001 ≤ P<0.01, *** for P<0.001. **(D)** Network diagram of the correlation between LVI-related differences in intestinal flora and immune cells. Each node represents each intestinal bacteria or immune cell, the node color represents the grouping, and the connecting line represents the existence of a significant correlation between two nodes; Pearson correlation coefficient values less than 0 (negative correlation) indicate the blue line, and Pearson correlation coefficient values greater than 0 (positive correlation) indicate the red line.

Tumor-infiltrating immune cells are an important component of the tumor microenvironment, involved in regulating the local tumor immune response, and are potential targets for tumor immunotherapy. To explore the relationship between LVI-related differential intestinal flora and tumor-infiltrating immune cells, we first produced strip plots demonstrating the composition of 22 infiltrating immune cells in 15 colorectal cancer patients with both information on vascular invasion and RNA sequencing (**Figure 3A**). The immune infiltration microenvironment of each patient was characterized by its own characteristics as seen in the figure. Overall, patients in the NLVI group had more plasma cells than in the LVI group, and patients in the LVI group had more B cells memory and B cells naive than in the NLVI group. Next, to investigate the association between LVI-related differential flora and immune cells, we correlated the dominant flora with 22 immune cells in the LVI and NLVI groups, respectively. In the LVI group, *f:Lachnospiraceae.g:Coprococcus*, *o:Lactobacillales.f:Lactobacillaceae* and *f:Lactobacillaceae.g:Lactobacillus* had a significant positive correlation with Macrophages, *g:Anaerovorax.s:uncultured_bacterium* has a significant positive correlation with Macrophages M2, *g:Bacteroides.s:Bacteroides_stercoris* and *o: Clostridiales.f:Ruminococcaceae* had a significant negative correlation with NK cells activated (**Figures 3B–D**). In the NLVI group, *f:Enterobacteriaceae.g:Enterobacter* showed a significant positive correlation with Dendritic cells resting and Macrophages M1 (**Figures 3C, D**). In summary, immune cell infiltration in CRC patients in the LVI and NLVI groups differed, and LVI-associated differential intestinal flora was significantly correlated with multiple immune cells, suggesting that LVI-associated differential intestinal flora may be involved in regulating immune cell infiltration in CRC.

### 3.6 Correlation of LVI-related differential intestinal flora with immune-related genes

**Figure 4:**
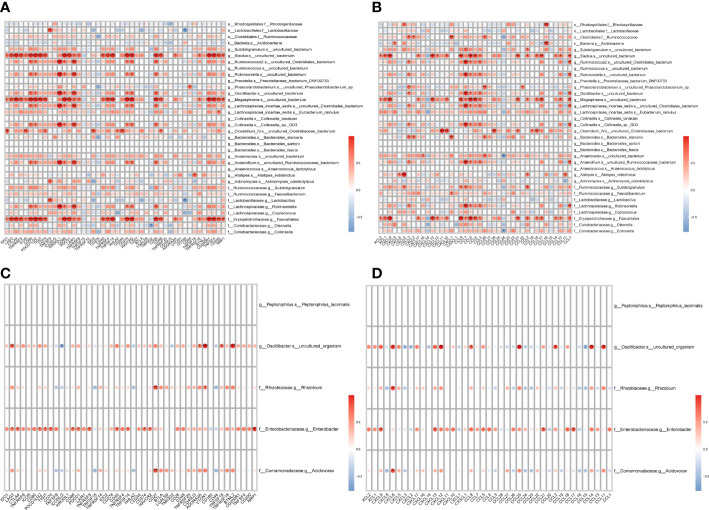
Correlation of LVI-related differential intestinal flora with immune-related genes **(A)** Heat map of correlation between the dominant flora and checkpoints in the LVI group. **(B)** Heat map of the correlation between the dominant flora and chemokines in the LVI group. **(C)** Heat map of correlation between dominant flora and checkpoints in NLVI group. **(D)** Heat map of the correlation between the dominant flora and chemokines in the NLVI group. The horizontal coordinates are genes, the vertical coordinates are bacteria, the red color in the graph represents positive correlation, the blue color represents negative correlation, the color depth represents the size of Pearson correlation coefficient, the color from light to dark indicates the value of Pearson correlation coefficient from small to large. The “*” in the graph indicates the P-value size: no * for P-value ≥ 0.05, * for 0.01 ≤ P<0.05, ** for 0.001 ≤ P<0.01, *** for P<0.001.

The immunity of the organism is closely related to the development of tumors. To investigate the relationship between LVI-associated differential gut flora and organismal immunity, we performed a correlation analysis between LVI-associated differential gut flora and common immune-related genes. Among the dominant bacteria in the LVI group, *g:Slackia.s:uncultured_bacterium*, *g:Megasphaera.s:uncultured_bacterium* and *f:Erysipelotrichaceae.g:Faecalitalea* were associated with multiple immune checkpoints (LAG3, CTLA4 and TNFRSF9 etc.) (as in **Figure 4A**), immune activating genes (CXCL12, CD86 and CD80 etc.) (as in **Supplementary Figure 2**), immune suppressor genes (CTLA4, CD96 and CD274 etc.) (as in **Supplementary Figure 3**), chemokines (CXCL9, CXCL13 and CCL5 etc.) (as in **Figure 4B**) and chemokine receptors (XCR1, CXCR3 and CCR5 etc.) (as in **Supplementary Figure 4**) all showed significant positive correlations. Among the dominant bacteria in the NLVI group, *g:Oscillibacter.s:uncultured_organism* and *f:Enterobacteriaceae.g:Enterobacter* were associated with multiple immune checkpoints (LAG3 and VTCN1 etc.) (as in **Figure 4C**), immune activation genes (TNFRSF17 and CD27 etc.) (as in **Supplementary Figure 5**), immunosuppressive genes (VTCN1 and TGFB1 etc.) (as in **Supplementary Figure 6**), chemokines (CXCL6 and CXCL9 etc.) (as in **Figure 4D**) and chemokine receptors (CCR1 and CCR2) (as in **Supplementary Figure 7**) showed significant correlations. The above results suggest that LVI-related differential intestinal flora may influence the expression of immune-related genes.

### 3.7 Identification of differential pathways and correlation between differential pathways and differential flora in colorectal cancer patients in the LVI and NLVI groups

**Figure 5:**
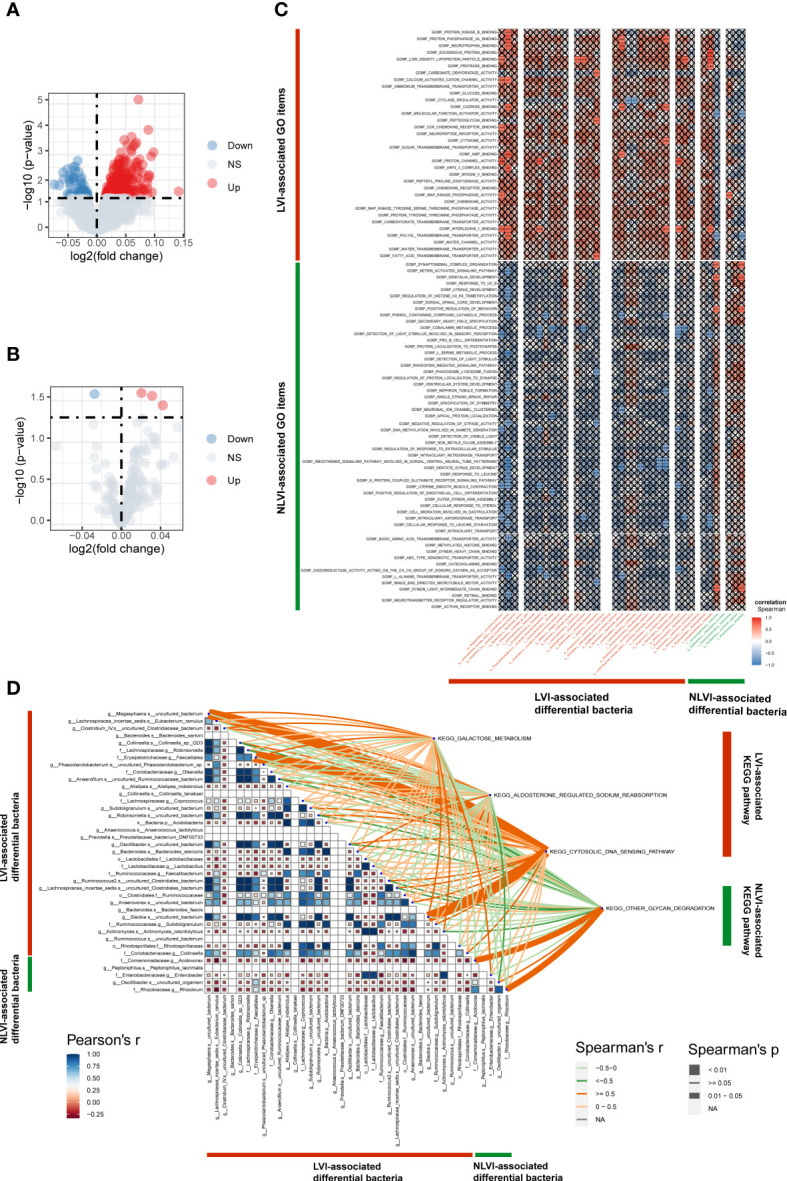
Identification of LVI-associated differential pathways and correlation of differential pathways with LVI-associated differential intestinal flora **(A)** GO volcano map of LVI-associated differential expression. **(B)** KEGG volcano plot of LVI-related differential expression. The horizontal coordinate indicates log2 (fold change), the farther the point is from the center, the greater the differential fold; the vertical coordinate indicates -log10 (p value), the closer the point to the top indicates the more significant expression difference. Each point represents a detected differentially expressed gene, red indicates an up-regulated gene, blue indicates a down-regulated gene, and gray indicates no differential gene. **(C)** Correlation graph of LVI-associated differential BP and MF with LVI-associated differential intestinal flora. Horizontal coordinates are bacteria, vertical coordinates are GO labels, red in the graph represents positive correlation, blue represents negative correlation, color depth represents Spearman’s correlation coefficient size, color from light to dark indicates Spearman’s correlation coefficient value from small to large. The “×” symbol in the graph indicates the P-value: with “×” symbol means P-value ≥ 0.05, without “×” symbol means P<0.05. **(D)** Correlation graph of LVI-associated differential KEGG with LVI-associated differential intestinal flora. The triangle graph represents the correlation graph of LVI-related differential in intestinal flora, the upper right corner is the KEGG label, the depth of the connecting color represents the magnitude of Spearman’s correlation coefficient r, the connecting line presented in dark orange indicates the correlation coefficient r≥0.05, representing a strong positive correlation. A light orange line indicates a correlation coefficient of 0<r<0.05, representing a weak positive correlation. A dark green line indicates a correlation coefficient r≥0.05, representing a strong negative correlation; a light green line indicates a correlation coefficient 0<r<0.05, representing a weak negative correlation; the thickness of the line represents the p-value, a thick line represents 0.01<P<0.05, and a thin line represents P≥0.05.

To explore LVI-related differential regulatory pathways and to investigate the potential impact of LVI-related differential gut flora on these pathways, we transformed RNA sequencing data from tumor samples of nine patients with gut flora 16sRNA sequencing data into corresponding scoring matrices by GO (Gene Ontology) and KEGG (Kyoto encyclopedia of Genes and Genomes) analysis through the ssGSEA method. GO enrichment analysis includes three aspects: biological process (BP), cellular component (CC), and molecular function (MF). Next, by differentially analyzing the GO and KEGG pathway score matrices of the LVI and NLVI groups, we found that a total of 327 GO pathways were significantly upregulated in the LVI group [GOMF_FATTY_ACID_TRANSMEMBRANE_TRANSPORTER_ACTIVITY (logFC=0.072,P<0.001) and GOBP_POLYOL_TRANSMEMBRANE_TRANSPORT (logFC=0.060,P<0.001) etc.]. And three KEGG pathways were significantly upregulated [KEGG_GALACTOSE_METABOLISM(logFC=0.021,P=0.025), KEGG_ALDOSTERONE_REGULATED_SODIUM_REABSORPTION(logFC=0.031,P=0.027) and KEGG_CYTOSOLIC_DNA_SENSING_PATHWAY(logFC=-0.042,P=0.036)]. There were 65 significantly upregulated GO pathways in the NLVI group [GOBP_INTRACILIARY_TRANSPORT(logFC=-0.037,P=0.003) and GOMF_NEUROTRANSMITTER_RECEPTOR_REGULATOR_ACTIVITY(logFC=- 0.074,P=0.015) etc.]. And one KEGG pathway was significantly upregulated [KEGG_OTHER_GLYCAN_DEGRADATION(logFC=-0.027,P=0.026)] (as shown in **Figures 5A, B**). The detailed GO enrichment list and KEGG pathway enrichment list are shown in **Supplementary Tables 5**, **6**, respectively. The above results suggest that colorectal cancer tissues with LVI are enriched for different biological functions.

Next, to investigate the relationship between LVI-associated genomic functions and LVI-associated differential intestinal flora, we used the colony counts of 39 LVI-associated differential flora from 9 patients to correlate with the LVI-associated BP, MF and KEGG pathway score matrices, respectively, and found significant correlations between some differential flora and some BP, MF and KEGG pathways. For example, GOMF_PROTEIN_PHOSPHA T ASE_2A_BINDING showed a significant strong positive correlation with *g:Lachnospiracea_incertae_sedis.s:Eubacterium_ramulus* (r=0.83,P<0.05) (**Figure 5C**; **Supplementary Table 7**). KEGG_CYTOSOLIC_DNA_SENSING_PATHWAY showed a significant strong positive correlation with *f:Erysipelotrichaceae.g:Faecalitalea* (r=0.84, P<0.01) (**Figure 5D**). These suggest that LVI-associated differential intestinal flora may influence vascular invasion in CRC through a number of potential biological functions.

### 3.8 Construction of gut microbiome signature for predicting LVI condition of CRC patients

**Figure 6:**
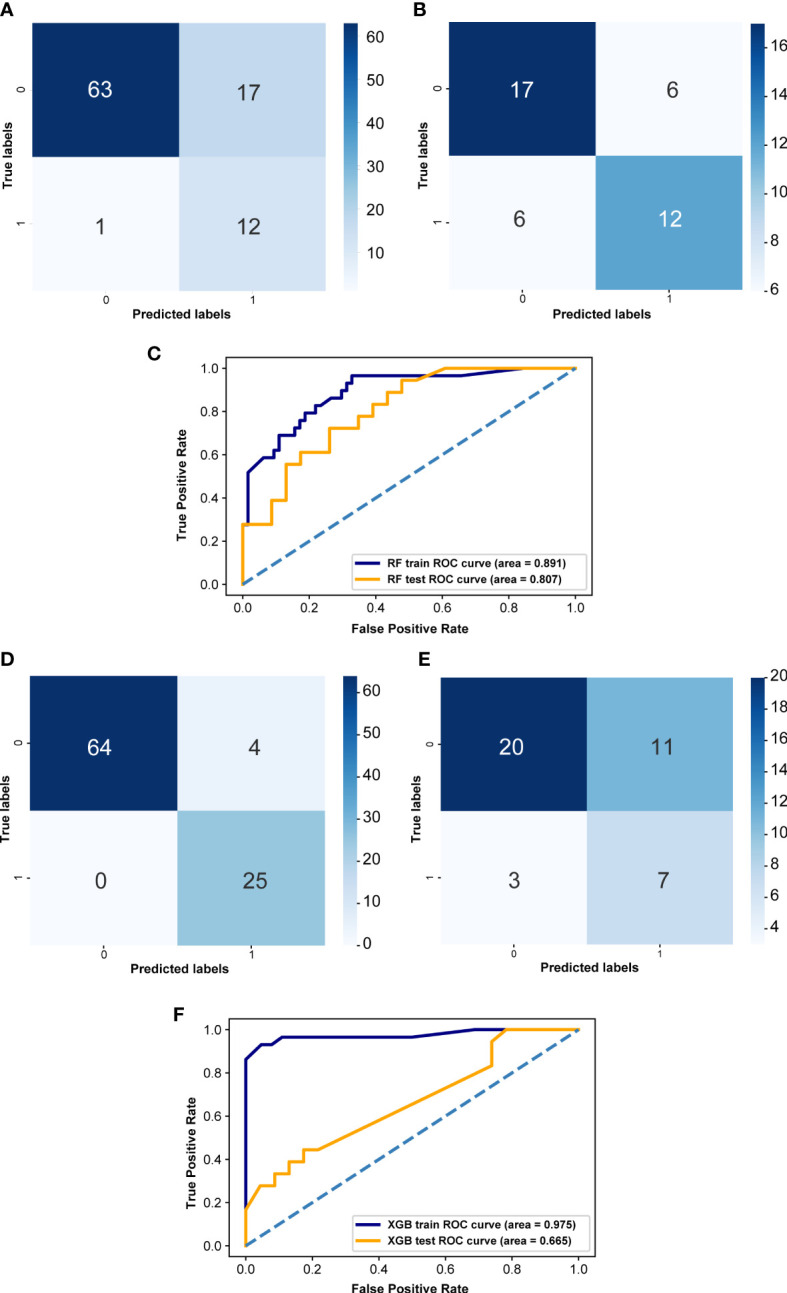
Effectiveness evaluation of RF and XGB prediction models **(A)** Confusion matrix of RF in the training set. **(B)** Confusion matrix of the RF model in the validation set. **(D)** Confusion matrix of XGB model in the training set. **(E)** Confusion matrix of XGB model in the validation set. X-axis represents the model prediction, y-axis represents the real situation, 1 represents correct prediction, 0 represents incorrect prediction, and the values in the box are the number of samples. **(C)** ROC curves of the random forest in both training set and validation set. **(F)** ROC curves of the XGB prediction model both training set and validation set. The horizontal coordinate is the false positive rate predicted by the model, the vertical coordinate indicates the true positive rate predicted by the model, and the area under the curve represents the AUC value, the higher the AUC value, the higher the diagnostic efficacy of the model.

To screen LVI-associated gut flora biomarkers and more accurately predict LVI in CRC patients, we constructed RF and XGB prediction models based on 39 LVI-associated differential gut flora obtained from LEfSe analysis. The confusion matrix of the training set of the RF-based LVI prediction model showed that the number of samples with a true negative (TN) prediction result was significantly higher than that of a false negative (FN), but the number of samples with a false positive (FP) result was higher than that of a true positive (TP) (**Figure 6A**), and the confusion matrix of the validation set showed that the number of samples with a true negative (TN) and true positive (TP) prediction result was higher than that of both false negative (FN) and false positive (FP) samples (**Figure 6B**), and the AUC value of the ROC curve for the training set was 0.891 and the AUC value of the ROC curve for the validation set was 0.807 (**Figure 6C**). The confusion matrix of the XGB-based LVI prediction model for the training set predicted significantly more samples as true negative (TN) and true positive (TP) than false negative (FN) and false positive (FP) (**Figure 6D**), and the confusion matrix of the validation set showed that the number of samples predicted as true negative (TN) was significantly higher than false negative (FN), but the number of false positive (FP) samples was more than the number of true number of positive (TP) samples (**Figure 6E**), the AUC value of the ROC curve for the training set was 0.975, and the AUC value of the ROC curve for the validation set was 0.665 (**Figure 6F**). In summary, although the confusion matrices of both models suggest a high false positive rate of model prediction. However, the AUC values of the ROC curves of the RF-based LVI prediction model were greater than 0.8 for both the training and validation sets. the AUC values of the ROC curves of the XGB-based LVI prediction model were greater than 0.95 for the training set, but less than 0.7 for the validation set. These results indicate that both models have some predictive accuracy and that the RF-based LVI prediction model has higher predictive efficacy, which can help guide the practice of predicting LVI in CRC patients by gut flora testing.

## 4 Discussion

In this study, we focused on exploring the differences between the structure and abundance of intestinal flora of CRC patients in the LVI and NLVI groups, and attempted to find microbial labels with predictive or discriminatory significance by analyzing their differential intestinal flora. We identified the relative abundance of each flora in the feces of CRC patients in LVI and NLVI groups by 16SrRNA sequencing technology and examined their biofunctional pathways, respectively.

We first compared the α-diversity and β-diversity of gut microorganisms in rectal cancer patients with LVI and NLVI nodes, and the results were not significantly different in either case, indicating that the number and composition of microbial community species did not differ significantly between the two sample groups. It has been shown that patients in the CRC group have more biodiversity in their gut flora than healthy subjects (Sheng et al., 2019; Zhang et al., 2021). This suggests that the occurrence of CRC may be closely related to the intestinal flora. It is worthwhile for us to investigate deeply whether there are certain flora in the intestinal flora of CRC patients that are associated with CRC-LVI. Therefore, we further identified the abundance of each intestinal community in both groups, looked for intestinal flora with significant differences in abundance between the two groups, and analyzed whether there was a correlation between them and CRC-LVI.

In the differential intestinal flora of the LVI and NLVI groups, *g:Alistipes.s:Alistipes_indistinctus* was closely correlated with other dominant bacteria. *Alistipes* is a Gram-negative bacterium of the phylum Mycobacterium, and a relatively new genus of bacteria, isolated mainly from medical clinical samples (Sakamoto et al., 2020). It has been suggested that *Alistipes* can act as a potential pathogen that may induce CRC (Parker et al., 2020). In addition, *Alistipes* has the most pathways of spoilage among commensal bacteria. Spoilage is the fermentation of undigested proteins in the gastrointestinal tract by the intestinal microbiota and usually results in the production of harmful metabolites by the bacteria. These products have been reported to be harmful and associated with CRC. Such products include ammonia, H2S, cresol, indole and phenol (Hughes et al., 2000). It has also been shown that *Alistipes_indistinctus* promotes inflammation and causes epithelial cell alterations, suggesting that this species is a potential driver of intestinal barrier dysfunction and inflammation (Kim et al., 2018). Impaired intestinal barrier function contributes to the pathogenesis of CRC. Intestinal barrier dysfunction can induce epithelial mesenchymal transformation and also allow harmful substances to enter the organism to induce chromosomal damage directly involved in tumorigenesis (Keating and Spencer, 2019). In addition, the impairment of tight junctions between cells and the defective immune barrier also promote the invasion and metastasis of CRC tumors (Li et al., 2020). Therefore, it is reasonable to assume that *Alistipes* can not only act as a potential pathogen to induce the development of CRC, but also it can promote the invasive and metastatic process of CRC tumors, and has a close relationship with CRC-LVI.

Analysis of the tumor microenvironment of immune cells showed that patients in the LVI group had more B cells memory and B cells naive than those in the NLVI group, and patients in the LVI group had less plasma cells than those in the NLVI group. It has been shown that tumor-infiltrating B cells can exert anti-tumor and pro-tumor effects, depending on their immunostimulatory or immunosuppressive activity, which varies by cancer type. B cells can protect against tumors under certain conditions, mainly by producing tumor-specific antibodies and presenting tumor antigens, but certain B cell subsets and antibody specificity can also suppress anti-cancer immunity and promote tumor growth (Sharonov et al., 2020; Kim et al., 2021). The higher B-cell content in the LVI group than in the NLVI group may be related to this. This is consistent with the results of our study. In the LVI group, there were some dominant colonies positively correlated with Macrophages M0 and Macrophages M2. In contrast, in the NLVI group, there were some dominant colonies that showed significant positive correlations with Macrophages M1. According to the study, Macrophages M0 and Macrophages M2 may play a major role in mediating immunosuppression and enhancing tumor aggressiveness (Najafi et al., 2019; Osman et al., 2020; Zheng et al., 2022). It has also been shown that M0 macrophages, M1 macrophages infiltrate significantly more in CRC compared to normal tissue. While M0 macrophages were highest in tumors with lymphatic invasion (Ge et al., 2019). In summary, we suggest that these dominant flora of the LVI group, which are positively correlated with Macrophages M0 and Macrophages M2, are closely related to CRC-LVI. These dominant flora can combine Macrophages M0 and Macrophages M2 to promote the CRC-LVI process to some extent.

We analyzed the correlation between differential intestinal flora and immune-related genes. Among the dominant bacteria in the LVI group, *g:Megasphaera.s:uncultured_bacterium* showed significant positive correlations with multiple immune checkpoints, immune activation genes, immune suppression genes, chemokines and chemokine receptors. Several studies have reported that the microbiome or its metabolites can enhance the antitumor effects of PD1 to some extent (Routy et al., 2018; Rossi et al., 2020). It has also been shown in studies that *Megasphaera* can significantly improve the antitumor efficacy of anti-PD1 therapy (Huang et al., 2022). In our study, a positive correlation between *Megasphaera* and multiple other immune checkpoints was also found, providing some research basis for the application of *Megasphaera* in antitumor immunotherapy. The predicted biofunctional analysis of gut microbes from CRC patients in the LVI and NLVI groups suggested significant differences in the abundance of KEGG pathways of microbial genes in the two groups of gut flora. Among the most significant pathways, Flagellar assembly and Bacterial chemotaxis may be associated with cancer development (Allali et al., 2018; Zhou et al., 2022). However, there is no direct evidence that they are directly related to LVI. How these differential KEGG pathways are linked to LVI remains to be proven.

RF model and XGB model are widely used machine learning methods with promising prediction results (AlThuwaynee et al., 2021; Hong et al., 2022; Yu JR. et al., 2022). We constructed a RF prediction model and an XGB prediction model using gut flora as a fecal microbial marker to distinguish CRC patients in the LVI and NLVI groups. The AUC values of both the training set ROC curve and the validation set ROC curve of the RF prediction model are greater than 0.8; the AUC value of the training set ROC curve of the XGB prediction model is 0.975, and the AUC value of the validation set ROC curve is 0.665. Both models have good diagnostic performance. Based on the machine learning model analysis, we further constructed the association between gut microbes and CRC-LVI, and demonstrated that the fecal biomarkers based on the dominant flora could be used to differentiate the LVI group from the NLVI group of CRC patients, which could help to identify potential microbial markers of gut flora for clinical application and to assess the LVI status of CRC patients easily and accurately.

There are also some shortcomings in this study: due to insufficient conditions, relevant basic experiments were not performed to verify the relationship between gut microorganisms and CRC-LCI. This study did not add the intestinal flora of healthy population as a control, and it was not possible to compare the differences between the flora of patients with various stages of CRC and healthy population, which was not conducive to the judgment of the benign and malignant degree of various enriched flora. In addition, the number of cases with both colorectal cancer tumor tissue samples and stool samples was too small, and the reliability of this study could also be improved if the number of such cases could be increased. In the follow-up study, we will try to overcome these shortcomings and strive to make better research results.

## 5 Conclusion

There were 39 intestinal flora with significantly different species abundance between LVI and NLVI groups, among which g:Alistipes.s:Alistipes_indistinctus with significantly higher species abundance in the LVI group was closely related to CRC-LVI, and Alistipes could promote the CRC-LVI process to some extent. LVI-associated differential gut flora is closely related to tumor-infiltrating immune cells and immune-related genes, and LVI-associated differential gut flora may be involved in regulating the infiltration of immune cells in CRC and, to some extent, influencing the expression of immune-related genes, which in turn affects the progression of CRC. colorectal cancer tissues with LVI are enriched for different biological functions, and LVI-associated differential gut flora may influence the process of vascular invasion in CRC through a number of potential biological functions. The RF prediction model and XGB prediction model constructed using intestinal flora as a fecal microbial marker have good diagnostic efficacy and can be used to predict CRC-LVI conditions.

## Data availability statement

The original contributions presented in the study are included in the article material. Further inquiries can be directed to the corresponding authors. The data presented in the study are deposited in the Nutstore repository, https://www.jianguoyun.com/p/DdAZBHQQsPGsChiC0YAFIAA, accession number is "hsnlmo".

## Ethics statement

This study was approved by the Ethics and Human Subject Committee of Guangxi Medical University Cancer Hospital.

## Author contributions

CC, KC, ZH, XH, XM, WT, JL: conceived and designed the experiments; JL, KC, XH, CC, BT, ZW, ZH, MQ, FH, XM, CL, WT:analyzed the data; JL, KC, XH, CC, ZW, ZH, MQ, FH, XM, CL, BT, WT: helped with reagents/materials/analysis tools; JL, XH, CC, ZW, ZH, MQ, FH, CL, KC, XM, BT, WT: contributed to the writing of the manuscript. All authors contributed to the article and approved the submitted version.

## Supplementary material

Supplementary Figure 1 Box plot of KEGG functional abundance in the LVI group versus the NLVI group of CRC patients.

Supplementary Figure 2 Heat map of the correlation between the dominant flora of LVI group and immune activation genes.

Supplementary Figure 3 Heat map of the correlation between the dominant flora of LVI group and immunosuppressive genes.

Supplementary Figure 4 Heat map of the correlation between the dominant flora of LVI group and chemokine receptors.

Supplementary Figure 5 Heat map of the correlation between the dominant flora of NLVI group and immune activation genes.

Supplementary Figure 6 Heat map of the correlation between the dominant flora of NLVI group and immunosuppressive genes.

Supplementary Figure 7 Heat map of the correlation between the dominant group of NLVI and chemokine receptors.

Supplementary Table 1 ADONIS test for Bray Distance of intestinal flora in CRC patients in the LVI and NLVI groups.

Supplementary Table 2 ADONIS test for Jaccard Distance of intestinal flora in CRC patients in the LVI and NLVI groups.

Supplementary Table 3 Results of LEfSe analysis.

Supplementary Table 4 KEGG functional pathways in the intestinal microbiome of CRC patients in the LVI and NLVI groups.

Supplementary Table 5 List of differential GO items of CRC patients stratified by LVI condition.

Supplementary Table 6 List of differential KEGG pathways of CRC patients stratified by LVI condition.

Supplementary Table 7 Correlation between enriched GO items of CRC patients in LVI group and LVI-associated differential intestinal microbiome.
