# Supplementary figures and images for: Identification of intestinal microbiome associated with lymph-vascular invasion in colorectal cancer patients and predictive label construction
Source: Front Cell Infect Microbiol. 2023 May 12;13:1098310. doi: 10.3389/fcimb.2023.1098310 (PMC10215531; doi:10.3389/fcimb.2023.1098310)

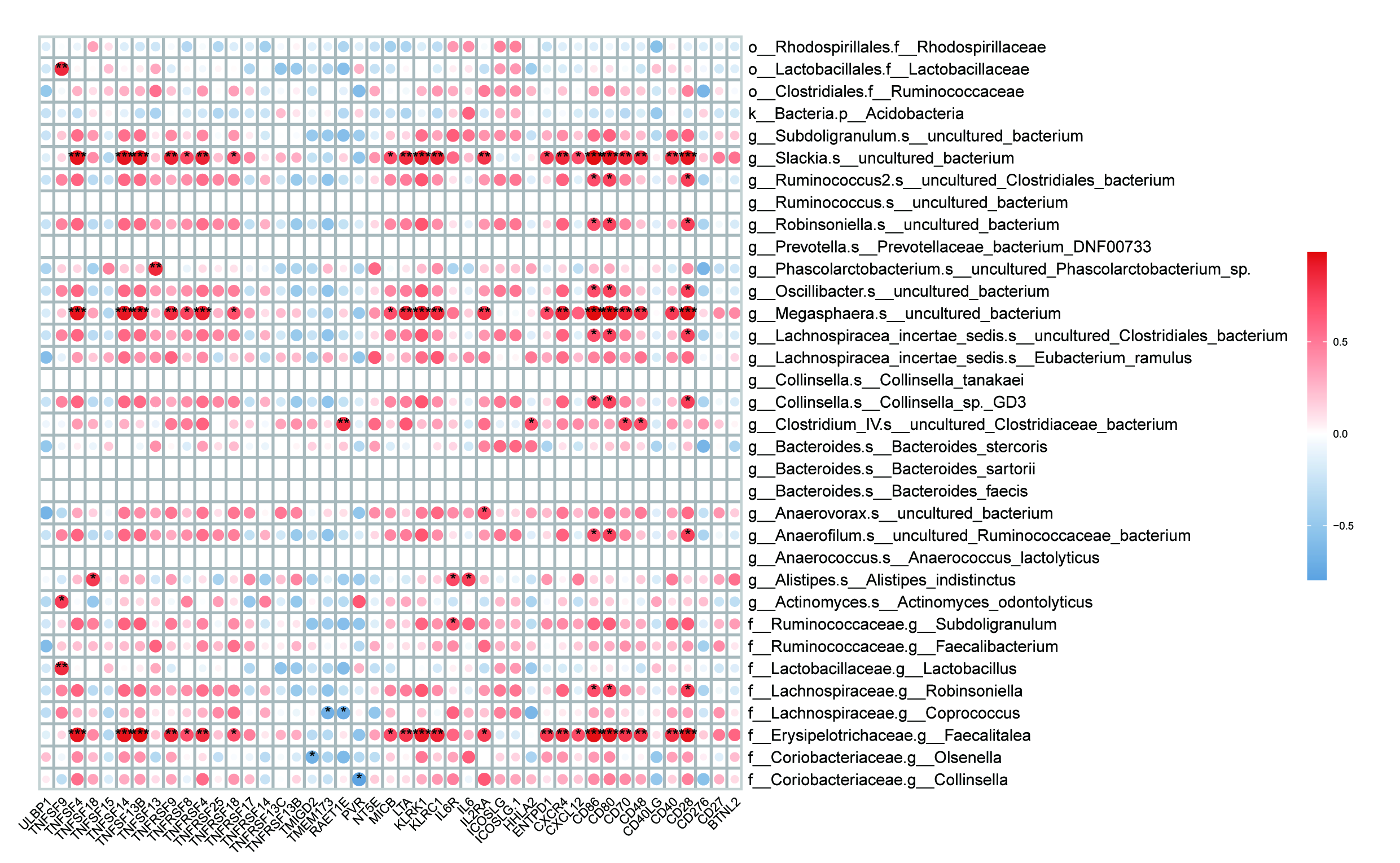

Supplement: Supplementary Figure 2 — Heat map of the correlation between the dominant flora of LVI group and immune activation genes. [file Image_2.tif]

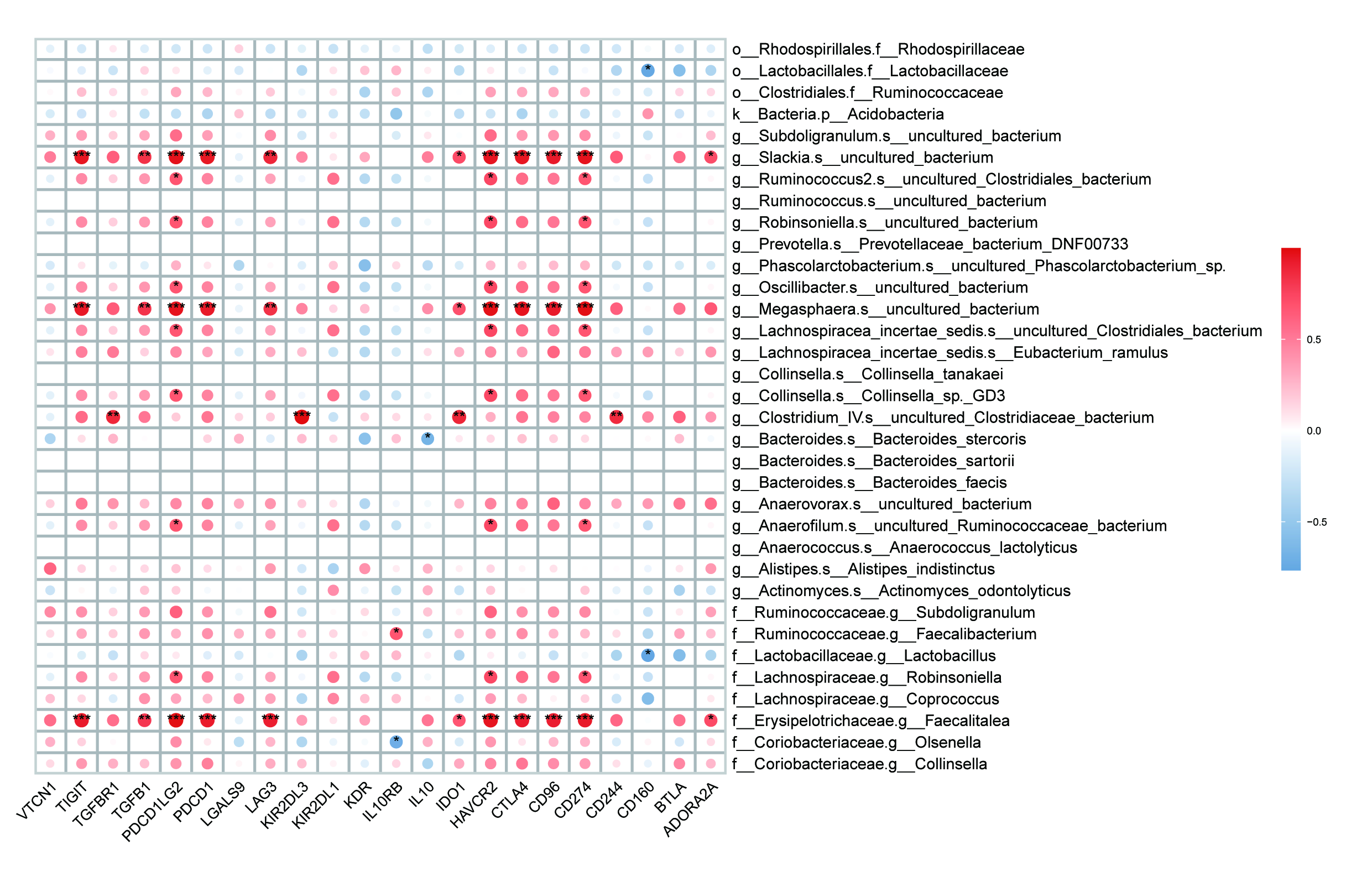

Supplement: Supplementary Figure 3 — Heat map of the correlation between the dominant flora of LVI group and immunosuppressive genes. [file Image_3.tif]

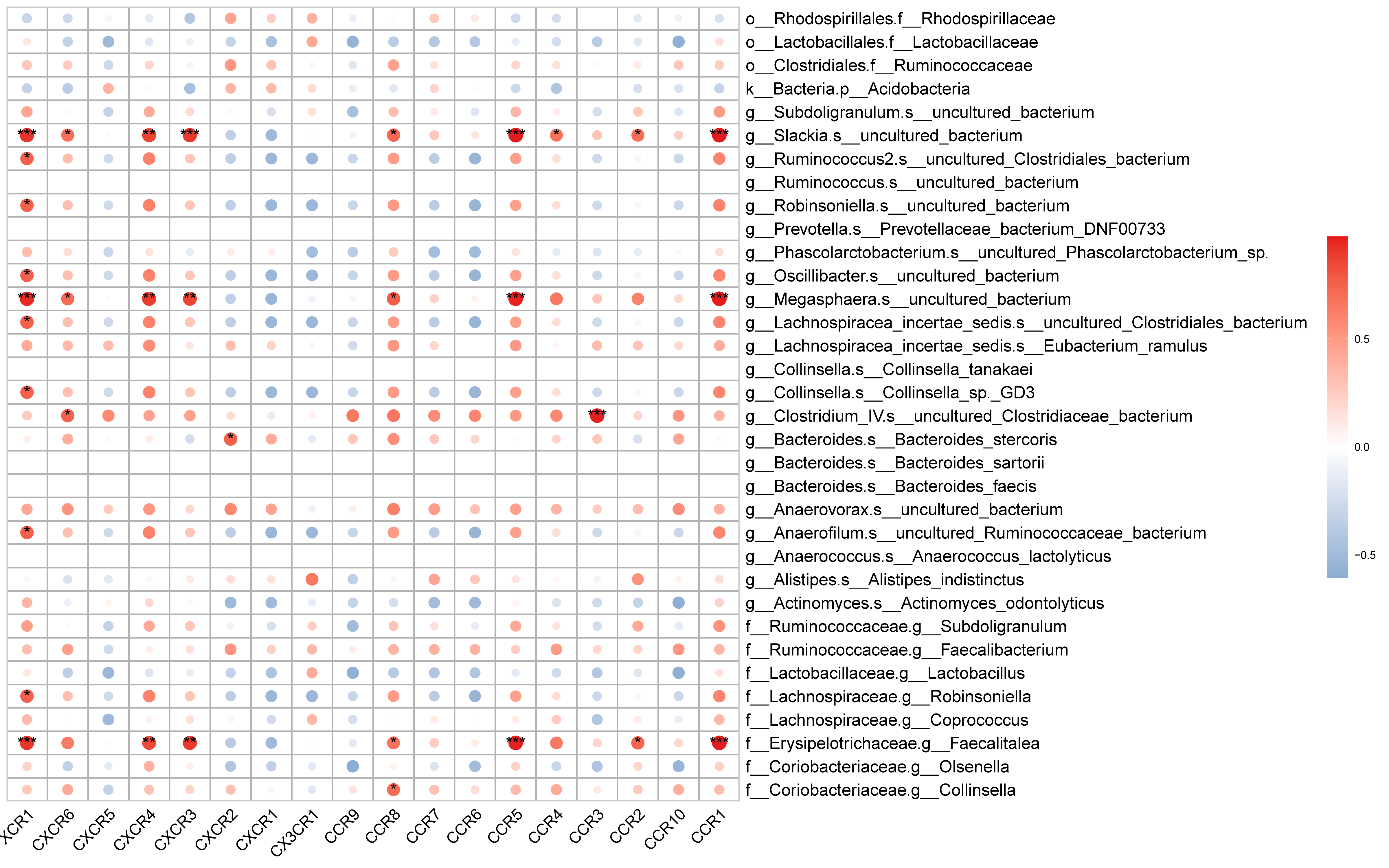

Supplement: Supplementary Figure 4 — Heat map of the correlation between the dominant flora of LVI group and chemokine receptors. [file Image_4.tif]

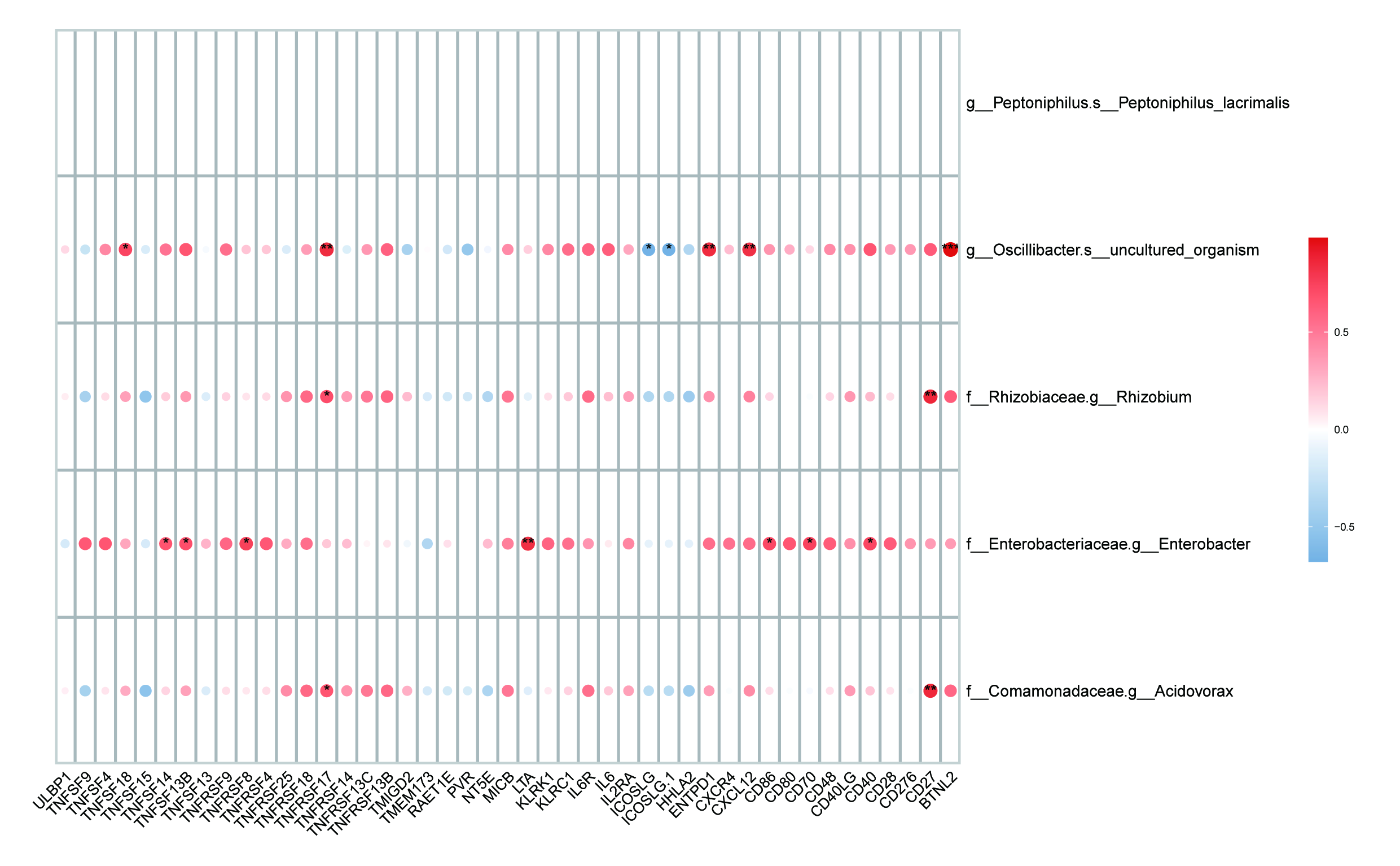

Supplement: Supplementary Figure 5 — Heat map of the correlation between the dominant flora of NLVI group and immune activation genes. [file Image_5.tif]

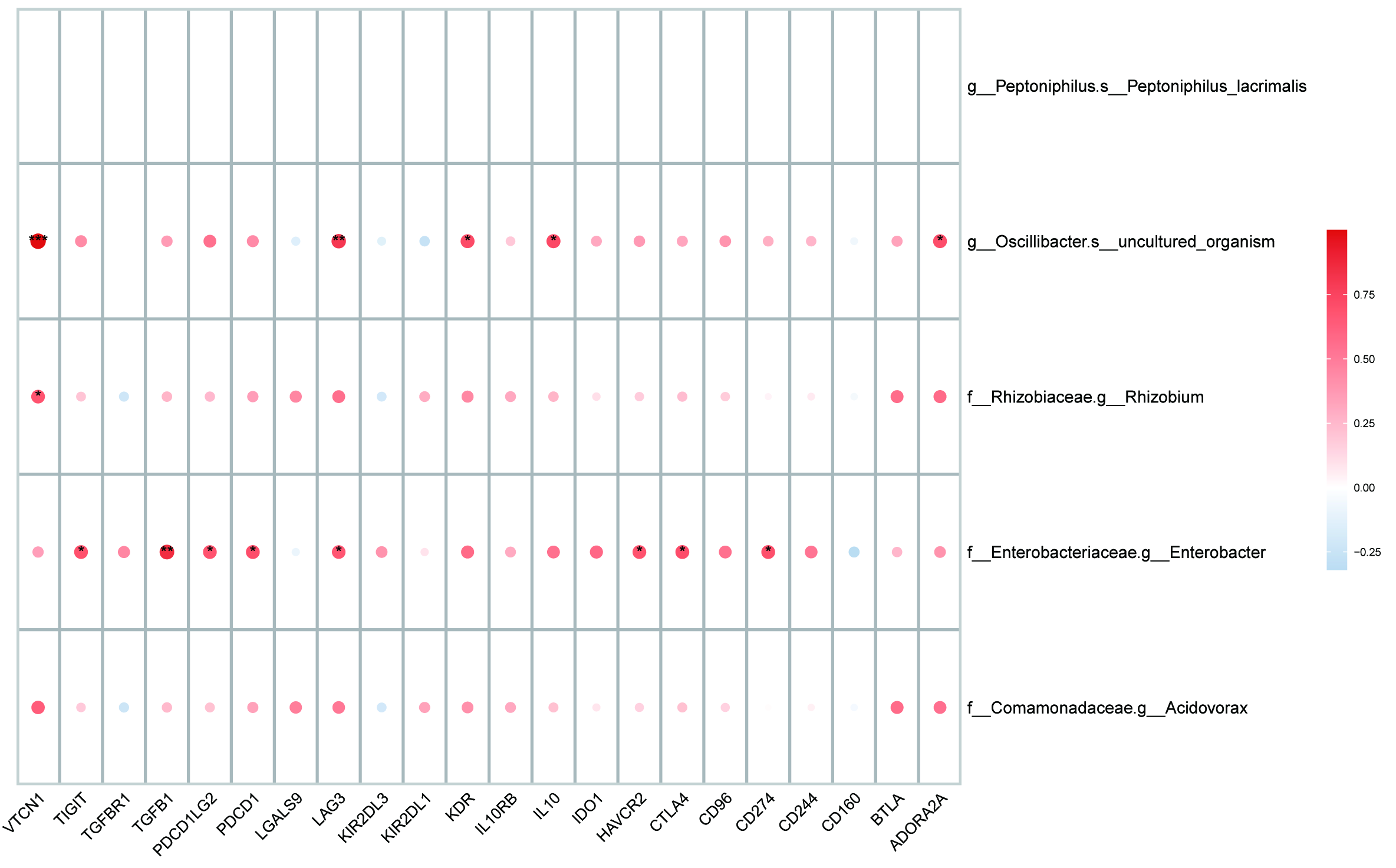

Supplement: Supplementary Figure 6 — Heat map of the correlation between the dominant flora of NLVI group and immunosuppressive genes. [file Image_6.tif]

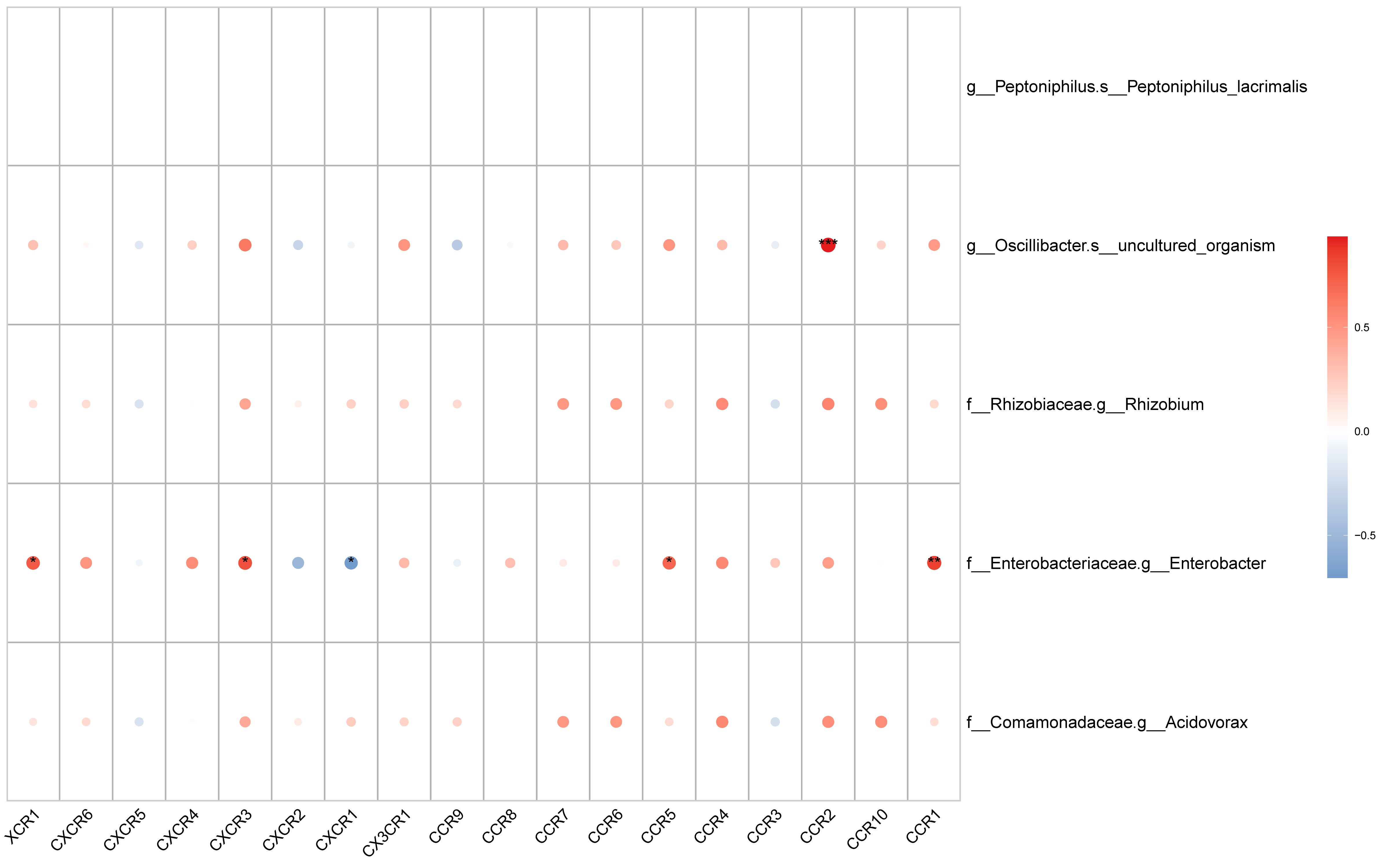

Supplement: Supplementary Figure 7 — Heat map of the correlation between the dominant group of NLVI and chemokine receptors. [file Image_7.tif]
