# Supplementary material for: Identification of intestinal microbiome associated with lymph-vascular invasion in colorectal cancer patients and predictive label construction
Source: Front Cell Infect Microbiol. 2023 May 12;13:1098310. doi: 10.3389/fcimb.2023.1098310 (PMC10215531; doi:10.3389/fcimb.2023.1098310)
Supplement: Supplementary Table 1 — ADONIS test for Bray Distance of intestinal flora in CRC patients in the LVI and NLVI groups. [file Table_1.docx]

**Supplementary Table 1. ADONIS test for Bray Distance of intestinal flora in CRC patients in the LVI and NLVI groups**

|  | Df | Sums Of Sqs | Mean Sqs | F.Model | R2 | Pr(>F) |
| --- | --- | --- | --- | --- | --- | --- |
| Group | 1 | 0.478733221 | 0.478733221 | 1.045508076 | 0.007858274 | 0.322 |
| Residuals | 132 | 60.44217788 | 0.457895287 |  | 0.992141726 |  |
| Total | 133 | 60.9209111 |  |  | 1 |  |
