# Supplementary material for: Identification of intestinal microbiome associated with lymph-vascular invasion in colorectal cancer patients and predictive label construction
Source: Front Cell Infect Microbiol. 2023 May 12;13:1098310. doi: 10.3389/fcimb.2023.1098310 (PMC10215531; doi:10.3389/fcimb.2023.1098310)
Supplement: Supplementary Table 2 — ADONIS test for Jaccard Distance of intestinal flora in CRC patients in the LVI and NLVI groups. [file Table_2.docx]

**Supplementary Table 2. ADONIS test for Jaccard Distance of intestinal flora in CRC patients in the LVI and NLVI groups**

|  | Df | SumsOfSqs | MeanSqs | F.Model | R2 | Pr(>F) |
| --- | --- | --- | --- | --- | --- | --- |
| Group | 1 | 0.467976544 | 0.467976544 | 1.086230233 | 0.008161853 | 0.1969 |
| Residuals | 132 | 56.86907058 | 0.430826292 |  | 0.991838147 |  |
| Total | 133 | 57.33704712 |  |  | 1 |  |
