# Supplementary material for: Identification of intestinal microbiome associated with lymph-vascular invasion in colorectal cancer patients and predictive label construction
Source: Front Cell Infect Microbiol. 2023 May 12;13:1098310. doi: 10.3389/fcimb.2023.1098310 (PMC10215531; doi:10.3389/fcimb.2023.1098310)
Supplement: Supplementary Table 3 — Results of LEfSe analysis. [file Table_3.docx]

| Taxonomy | Group | LDA (log10) | P value |
| --- | --- | --- | --- |
| g__Megasphaera.s__uncultured_bacterium | LVI | 2.761897 | 0.002184 |
| g__Lachnospiracea_incertae_sedis.s__Eubacterium_ramulus | LVI | 3.875302 | 0.00499 |
| g__Clostridium_IV.s__uncultured_Clostridiaceae_bacterium | LVI | 3.773704 | 0.005279 |
| g__Bacteroides.s__Bacteroides_sartorii | LVI | 4.955067 | 0.005925 |
| g__Collinsella.s__Collinsella_sp__GD3 | LVI | 3.522164 | 0.005925 |
| f__Lachnospiraceae.g__Robinsoniella | LVI | 3.05836 | 0.006663 |
| f__Erysipelotrichaceae.g__Faecalitalea | LVI | 3.279735 | 0.006827 |
| g__Phascolarctobacterium.s__uncultured_Phascolarctobacterium_sp_ | LVI | 2.399343 | 0.007177 |
| f__Coriobacteriaceae.g__Olsenella | LVI | 3.82532 | 0.008901 |
| g__Anaerofilum.s__uncultured_Ruminococcaceae_bacterium | LVI | 3.235937 | 0.010567 |
| f__Comamonadaceae.g__Acidovorax | NLVI | 2.869756 | 0.011347 |
| g__Alistipes.s__Alistipes_indistinctus | LVI | 3.23498 | 0.012749 |
| g__Collinsella.s__Collinsella_tanakaei | LVI | 3.235672 | 0.014253 |
| f__Lachnospiraceae.g__Coprococcus | LVI | 4.303934 | 0.014654 |
| g__Subdoligranulum.s__uncultured_bacterium | LVI | 3.921094 | 0.015628 |
| g__Robinsoniella.s__uncultured_bacterium | LVI | 2.805488 | 0.017584 |
| k__Bacteria.p__Acidobacteria | LVI | 3.267791 | 0.017584 |
| g__Anaerococcus.s__Anaerococcus_lactolyticus | LVI | 2.215837 | 0.017584 |
| g__Prevotella.s__Prevotellaceae_bacterium_DNF00733 | LVI | 2.539865 | 0.017584 |
| g__Oscillibacter.s__uncultured_bacterium | LVI | 3.668498 | 0.017584 |
| g__Peptoniphilus.s__Peptoniphilus_lacrimalis | NLVI | 2.75165 | 0.022147 |
| g__Bacteroides.s__Bacteroides_stercoris | LVI | 4.565994 | 0.022251 |
| o__Lactobacillales.f__Lactobacillaceae | LVI | 5.517003 | 0.02289 |
| f__Lactobacillaceae.g__Lactobacillus | LVI | 5.516707 | 0.023601 |
| f__Ruminococcaceae.g__Faecalibacterium | LVI | 4.904693 | 0.027587 |
| f__Enterobacteriaceae.g__Enterobacter | NLVI | 3.26483 | 0.031942 |
| g__Ruminococcus2.s__uncultured_Clostridiales_bacterium | LVI | 3.071165 | 0.033771 |
| g__Lachnospiracea_incertae_sedis.s__uncultured_Clostridiales_bacterium | LVI | 2.536557 | 0.034731 |
| o__Clostridiales.f__Ruminococcaceae | LVI | 5.294661 | 0.03569 |
| g__Anaerovorax.s__uncultured_bacterium | LVI | 3.500808 | 0.038269 |
| g__Bacteroides.s__Bacteroides_faecis | LVI | 2.934526 | 0.040407 |
| g__Slackia.s__uncultured_bacterium | LVI | 2.677166 | 0.041724 |
| g__Oscillibacter.s__uncultured_organism | NLVI | 2.281033 | 0.041789 |
| f__Ruminococcaceae.g__Subdoligranulum | LVI | 3.915407 | 0.041951 |
| g__Actinomyces.s__Actinomyces_odontolyticus | LVI | 2.205955 | 0.042828 |
| g__Ruminococcus.s__uncultured_bacterium | LVI | 3.067829 | 0.042946 |
| o__Rhodospirillales.f__Rhodospirillaceae | LVI | 3.05322 | 0.043123 |
| f__Coriobacteriaceae.g__Collinsella | LVI | 4.781659 | 0.043143 |
| f__Rhizobiaceae.g__Rhizobium | NLVI | 3.678794 | 0.046689 |

**Supplementary Table 3. Results of LEfSe analysis**
