# Supplementary material for: Identification of intestinal microbiome associated with lymph-vascular invasion in colorectal cancer patients and predictive label construction
Source: Front Cell Infect Microbiol. 2023 May 12;13:1098310. doi: 10.3389/fcimb.2023.1098310 (PMC10215531; doi:10.3389/fcimb.2023.1098310)
Supplement: Supplementary Table 4 — KEGG functional pathways in the intestinal microbiome of CRC patients in the LVI and NLVI groups. [file Table_4.docx]

**Supplementary Table 4. KEGG functional pathways in the intestinal microbiome of CRC patients in the LVI and NLVI groups**

| KEGG_pathway | Mean In NLVI | Mean In LVI | P value |
| --- | --- | --- | --- |
| ko00621:Dioxin degradation | 237188084.2 | 406755655.7 | 0.003621516 |
| ko02060:Phosphotransferase system (PTS) | 459614063.6 | 766660748.8 | 0.008090304 |
| ko00930:Caprolactam degradation | 37164423.85 | 66102745.42 | 0.012783154 |
| ko02040:Flagellar assembly | 572882954.6 | 621305375.6 | 0.012951812 |
| ko00072:Synthesis and degradation of ketone bodies | 354389537.7 | 489640891.6 | 0.013122436 |
| ko02030:Bacterial chemotaxis | 979968570.3 | 1137307652 | 0.014749708 |
| ko00361:Chlorocyclohexane and chlorobenzene degradation | 89904405.7 | 115766462.3 | 0.016552277 |
| ko04020:Calcium signaling pathway | 537.5974954 | 86036.67104 | 0.016641392 |
| ko03450:Non-homologous end-joining | 22706957.52 | 21630798.56 | 0.01785893 |
| ko05144:Malaria | 0 | 34581.50789 | 0.018022064 |
| ko00633:Nitrotoluene degradation | 419348914.1 | 491875836 | 0.018779455 |
| ko00362:Benzoate degradation | 204974890.2 | 251345754 | 0.020238029 |
| ko00311:Penicillin and cephalosporin biosynthesis | 37599082.51 | 59641191.38 | 0.020745714 |
| ko02020:Two-component system | 441680597.5 | 505455716.1 | 0.021528055 |
| ko00601:Glycosphingolipid biosynthesis - lacto and neolacto series | 288832.3459 | 506410.9061 | 0.029513776 |
| ko04080:Neuroactive ligand-receptor interaction | 0.010714943 | 12.00617234 | 0.030905014 |
| ko05143:African trypanosomiasis | 6824204.495 | 12346570.5 | 0.032344842 |
| ko00561:Glycerolipid metabolism | 648807249.4 | 727594235.7 | 0.03272778 |
| ko04122:Sulfur relay system | 1357029091 | 1460873320 | 0.033110194 |
| ko01040:Biosynthesis of unsaturated fatty acids | 493064440.6 | 526246422.9 | 0.033496426 |
| ko02010:ABC transporters | 884020108.3 | 979490143.1 | 0.033496426 |
| ko05100:Bacterial invasion of epithelial cells | 10040079.94 | 18740167.92 | 0.033886506 |
| ko05111:Vibrio cholerae pathogenic cycle | 210755605.4 | 211865206.9 | 0.033886506 |
| ko03013:RNA transport | 73656492.5 | 76977972.71 | 0.034280463 |
| ko01051:Biosynthesis of ansamycins | 5773177045 | 6171154680 | 0.0354859 |
| ko00791:Atrazine degradation | 88032649.02 | 133144235.7 | 0.036726378 |
| ko00350:Tyrosine metabolism | 355809330.9 | 390667444.4 | 0.036727316 |
| ko00440:Phosphonate and phosphinate metabolism | 186152956.2 | 217282587.7 | 0.038878543 |
| ko00051:Fructose and mannose metabolism | 1530473408 | 1535297270 | 0.039321397 |
| ko00564:Glycerophospholipid metabolism | 818622939.4 | 816578074.2 | 0.039768526 |
| ko00052:Galactose metabolism | 1700529898 | 1754238416 | 0.04160043 |
| ko00360:Phenylalanine metabolism | 411133741 | 419908354.9 | 0.04160043 |
| ko00061:Fatty acid biosynthesis | 2210912652 | 2233412540 | 0.042069412 |
| ko00312:beta-Lactam resistance | 308201445.5 | 420931539.8 | 0.042069412 |
| ko00680:Methane metabolism | 747626237.9 | 735059017.9 | 0.043990322 |
| ko04626:Plant-pathogen interaction | 262421036.1 | 251220132.8 | 0.044978219 |
| ko00627:Aminobenzoate degradation | 193853053.3 | 189535830.7 | 0.045479143 |
| ko00650:Butanoate metabolism | 945811268.6 | 938817804.5 | 0.045479143 |
| ko00300:Lysine biosynthesis | 2190068138 | 2183714388 | 0.046495102 |
| ko00010:Glycolysis / Gluconeogenesis | 1597929694 | 1587334123 | 0.047530099 |
| ko00620:Pyruvate metabolism | 1638389403 | 1625095170 | 0.048054819 |
| ko00473:D-Alanine metabolism | 2432198808 | 2292522400 | 0.049118869 |
| ko00910:Nitrogen metabolism | 891248425.3 | 866116603.8 | 0.049118869 |
| ko00500:Starch and sucrose metabolism | 1586232062 | 1653062919 | 0.049658265 |
| ko00550:Peptidoglycan biosynthesis | 2656258719 | 2471221207 | 0.049658265 |
| ko00920:Sulfur metabolism | 1117117875 | 1116528559 | 0.049658265 |
| ko03070:Bacterial secretion system | 1104077943 | 1039650702 | 0.049658265 |
| ko00053:Ascorbate and aldarate metabolism | 368221042.1 | 390827039.3 | 0.050202621 |
| ko00030:Pentose phosphate pathway | 2320853805 | 2281665564 | 0.050751969 |
| ko00471:D-Glutamine and D-glutamate metabolism | 2945367690 | 2762226822 | 0.051306344 |
| ko00290:Valine, leucine and isoleucine biosynthesis | 3075360506 | 2982628275 | 0.05186578 |
| ko00514:Other types of O-glycan biosynthesis | 245024.6542 | 207265.4167 | 0.052235335 |
| ko00630:Glyoxylate and dicarboxylate metabolism | 958267764.5 | 916031820 | 0.05243031 |
| ko00900:Terpenoid backbone biosynthesis | 1865061869 | 1698444819 | 0.05243031 |
| ko03010:Ribosome | 2283881261 | 2055138687 | 0.05243031 |
| ko00270:Cysteine and methionine metabolism | 1746993337 | 1742325653 | 0.052999968 |
| ko00970:Aminoacyl-tRNA biosynthesis | 2346832321 | 2178730250 | 0.052999968 |
| ko03008:Ribosome biogenesis in eukaryotes | 78324269.54 | 73681076.33 | 0.052999968 |
| ko03060:Protein export | 2071610621 | 1904163805 | 0.052999968 |
| ko00230:Purine metabolism | 1274313691 | 1197235283 | 0.053574788 |
| ko04112:Cell cycle - Caulobacter | 2189017921 | 1999858189 | 0.053574788 |
| ko00760:Nicotinate and nicotinamide metabolism | 1624401924 | 1430103972 | 0.054154805 |
| ko00770:Pantothenate and CoA biosynthesis | 2554715041 | 2355642354 | 0.054154805 |
| ko03410:Base excision repair | 1337140045 | 1197039971 | 0.054154805 |
| ko00250:Alanine, aspartate and glutamate metabolism | 2434522374 | 2292385585 | 0.054740053 |
| ko00330:Arginine and proline metabolism | 1028222259 | 995492870.3 | 0.054740053 |
| ko00640:Propanoate metabolism | 935544245.4 | 917320916.8 | 0.054740053 |
| ko00710:Carbon fixation in photosynthetic organisms | 2296994900 | 2086367275 | 0.054740053 |
| ko00740:Riboflavin metabolism | 1301120551 | 1063949396 | 0.054740053 |
| ko03420:Nucleotide excision repair | 1174272783 | 1062878226 | 0.055330566 |
| ko03440:Homologous recombination | 2177931720 | 1996562526 | 0.055330566 |
| ko00071:Fatty acid metabolism | 473886988.9 | 477542356.1 | 0.055926379 |
| ko00240:Pyrimidine metabolism | 1710590232 | 1536502794 | 0.055926379 |
| ko00430:Taurine and hypotaurine metabolism | 980197596.9 | 896972962.3 | 0.055926379 |
| ko00520:Amino sugar and nucleotide sugar metabolism | 1665312021 | 1561883021 | 0.056527526 |
| ko00730:Thiamine metabolism | 2323713915 | 2180874090 | 0.056527526 |
| ko03030:DNA replication | 1786781946 | 1629570805 | 0.056527526 |
| ko00040:Pentose and glucuronate interconversions | 981910113.6 | 989753435.4 | 0.057134041 |
| ko03430:Mismatch repair | 2480599569 | 2269639508 | 0.057134041 |
| ko00260:Glycine, serine and threonine metabolism | 1553653249 | 1435592172 | 0.05774596 |
| ko03020:RNA polymerase | 1506626921 | 1398378657 | 0.05774596 |
| ko00121:Secondary bile acid biosynthesis | 1546320787 | 1717993009 | 0.058363317 |
| ko00480:Glutathione metabolism | 648865885.6 | 632856331.2 | 0.058363317 |
| ko00750:Vitamin B6 metabolism | 1666916736 | 1439107600 | 0.058986146 |
| ko00941:Flavonoid biosynthesis | 12343827.37 | 13410836.14 | 0.058986146 |
| ko00120:Primary bile acid biosynthesis | 172655696 | 191170600.7 | 0.060248363 |
| ko04910:Insulin signaling pathway | 135048020.1 | 124884328.2 | 0.060248363 |
| ko00450:Selenocompound metabolism | 1557565535 | 1469267293 | 0.06088782 |
| ko00860:Porphyrin and chlorophyll metabolism | 963837593.8 | 884973990.7 | 0.06088782 |
| ko00960:Tropane, piperidine and pyridine alkaloid biosynthesis | 559162438.1 | 528459347.9 | 0.06153289 |
| ko00340:Histidine metabolism | 1699200343 | 1599038735 | 0.062840009 |
| ko00130:Ubiquinone and other terpenoid-quinone biosynthesis | 689220523.4 | 490504822 | 0.063502128 |
| ko00380:Tryptophan metabolism | 249816671.3 | 223237234.9 | 0.063832032 |
| ko00670:One carbon pool by folate | 2618842442 | 2288049135 | 0.06417 |
| ko00660:C5-Branched dibasic acid metabolism | 2461956138 | 2392021444 | 0.064843661 |
| ko03018:RNA degradation | 925910252.5 | 802751091 | 0.064843661 |
| ko04141:Protein processing in endoplasmic reticulum | 73584453.13 | 58330938.72 | 0.064843661 |
| ko00190:Oxidative phosphorylation | 698135702.1 | 619538398.5 | 0.066208491 |
| ko00310:Lysine degradation | 261456979.4 | 224077989 | 0.069724355 |
| ko00400:Phenylalanine, tyrosine and tryptophan biosynthesis | 1697710946 | 1578410505 | 0.070445605 |
| ko00720:Carbon fixation pathways in prokaryotes | 1727992434 | 1416478584 | 0.070445605 |
| ko00562:Inositol phosphate metabolism | 292795623.2 | 287215914.6 | 0.074902652 |
| ko05120:Epithelial cell signaling in Helicobacter pylori infection | 238848990.5 | 216100687.3 | 0.074902652 |
| ko00521:Streptomycin biosynthesis | 2676812492 | 2365491949 | 0.07721607 |
| ko01055:Biosynthesis of vancomycin group antibiotics | 3585161114 | 3122555897 | 0.07721607 |
| ko00472:D-Arginine and D-ornithine metabolism | 120581952.9 | 149240258.4 | 0.07958738 |
| ko04144:Endocytosis | 549126.7344 | 857567.7834 | 0.085343246 |
| ko00020:Citrate cycle (TCA cycle) | 1613426161 | 1277980811 | 0.085351039 |
| ko00780:Biotin metabolism | 2372815124 | 1936509172 | 0.085351039 |
| ko00906:Carotenoid biosynthesis | 20441234.63 | 13358209.89 | 0.087058395 |
| ko00908:Zeatin biosynthesis | 1053553778 | 821847929.6 | 0.088793213 |
| ko00790:Folate biosynthesis | 1944976314 | 1516971843 | 0.09055578 |
| ko04614:Renin-angiotensin system | 6322.194125 | 0 | 0.09664958 |
| ko00280:Valine, leucine and isoleucine degradation | 597243190 | 478507160.9 | 0.096947448 |
| ko04146:Peroxisome | 331426722.6 | 275503480.6 | 0.107715007 |
| ko00785:Lipoic acid metabolism | 1901415178 | 1116365422 | 0.110810315 |
| ko05150:Staphylococcus aureus infection | 35493892.14 | 57966767.5 | 0.11291258 |
| ko01053:Biosynthesis of siderophore group nonribosomal peptides | 135172967.7 | 88813495.32 | 0.116124764 |
| ko04621:NOD-like receptor signaling pathway | 136995923.9 | 103219876.5 | 0.116124764 |
| ko00600:Sphingolipid metabolism | 856261931.2 | 702896085.7 | 0.121637477 |
| ko00965:Betalain biosynthesis | 39326.71638 | 18080.91117 | 0.124715825 |
| ko00540:Lipopolysaccharide biosynthesis | 1325127067 | 838603966.5 | 0.134484383 |
| ko00643:Styrene degradation | 37334789.86 | 78896979.35 | 0.139102018 |
| ko03050:Proteasome | 2196655.357 | 3748372.609 | 0.157736424 |
| ko00626:Naphthalene degradation | 110483114.8 | 126562288.2 | 0.157801974 |
| ko04970:Salivary secretion | 40481.23487 | 446287.4475 | 0.158463386 |
| ko00511:Other glycan degradation | 3183061422 | 2267988513 | 0.161891502 |
| ko00140:Steroid hormone biosynthesis | 105737128.1 | 69032820.47 | 0.185456939 |
| ko00531:Glycosaminoglycan degradation | 1488233764 | 844917646 | 0.198150358 |
| ko00363:Bisphenol degradation | 92463196.97 | 40874283.88 | 0.242008303 |
| ko05146:Amoebiasis | 21797980.48 | 16514979.21 | 0.283555691 |
| ko00983:Drug metabolism - other enzymes | 321260110.1 | 291599131 | 0.29458429 |
| ko00510:N-Glycan biosynthesis | 83447742.27 | 58911084.65 | 0.300637962 |
| ko00513:Various types of N-glycan biosynthesis | 3471.421379 | 0 | 0.301953812 |
| ko04512:ECM-receptor interaction | 5299.603038 | 0 | 0.301953812 |
| ko04142:Lysosome | 25688381.95 | 369007.4873 | 0.318413457 |
| ko05012:Parkinson's disease | 2441526.63 | 1767395.136 | 0.331923322 |
| ko04210:Apoptosis | 65858901.71 | 34292373.22 | 0.334480021 |
| ko00591:Linoleic acid metabolism | 221922973.2 | 180242071.8 | 0.345525729 |
| ko00100:Steroid biosynthesis | 35184091.9 | 14787767.97 | 0.370683622 |
| ko05322:Systemic lupus erythematosus | 61778.08637 | 56213.68779 | 0.399563928 |
| ko00943:Isoflavonoid biosynthesis | 29666.37138 | 12844.27584 | 0.411231147 |
| ko00830:Retinol metabolism | 57357523.27 | 49970042.71 | 0.445146129 |
| ko05142:Chagas disease (American trypanosomiasis) | 209761.4194 | 20251.27901 | 0.460293532 |
| ko04974:Protein digestion and absorption | 90143440.56 | 49615242.32 | 0.472745605 |
| ko00623:Toluene degradation | 72924761.54 | 106816178 | 0.502808278 |
| ko00524:Butirosin and neomycin biosynthesis | 129532736.5 | 133767985.1 | 0.518573613 |
| ko04310:Wnt signaling pathway | 17.97888736 | 90.20690426 | 0.521829224 |
| ko04110:Cell cycle | 16816.37953 | 1925.055647 | 0.524001874 |
| ko00625:Chloroalkane and chloroalkene degradation | 273743412.6 | 223760408.5 | 0.527075442 |
| ko00980:Metabolism of xenobiotics by cytochrome P450 | 13149591.3 | 21361949.13 | 0.585083352 |
| ko00196:Photosynthesis - antenna proteins | 29040.65713 | 82378.27356 | 0.595593252 |
| ko00410:beta-Alanine metabolism | 511771114.8 | 422895525.2 | 0.600728922 |
| ko00622:Xylene degradation | 37391453.76 | 98665000.44 | 0.67250058 |
| ko00903:Limonene and pinene degradation | 66914962.35 | 59501539.37 | 0.673512173 |
| ko04962:Vasopressin-regulated water reabsorption | 16427.98783 | 21511.71615 | 0.680618653 |
| ko00624:Polycyclic aromatic hydrocarbon degradation | 448151.778 | 693474.6837 | 0.701269159 |
| ko05410:Hypertrophic cardiomyopathy (HCM) | 1103835.781 | 750552.987 | 0.715651514 |
| ko01057:Biosynthesis of type II polyketide products | 37414.57184 | 11578.91489 | 0.725840961 |
| ko00642:Ethylbenzene degradation | 38164609.91 | 75977571.1 | 0.739307208 |
| ko04075:Plant hormone signal transduction | 250678.8047 | 455581.3309 | 0.756386293 |
| ko00364:Fluorobenzoate degradation | 12200592.94 | 24711140.29 | 0.777668245 |
| ko03040:Spliceosome | 66625.91063 | 28365.55806 | 0.777671344 |
| ko00195:Photosynthesis | 75827893.19 | 114000873.7 | 0.781291842 |
| ko00460:Cyanoamino acid metabolism | 255379680.7 | 375151129.1 | 0.79037403 |
| ko05110:Vibrio cholerae infection | 55406.12055 | 46294.61736 | 0.813196331 |
| ko00523:Polyketide sugar unit biosynthesis | 118488763.2 | 189713811.9 | 0.840973256 |
| ko05145:Toxoplasmosis | 80739.18198 | 175273.1643 | 0.867761464 |
| ko05130:Pathogenic Escherichia coli infection | 20171.48971 | 61016.17021 | 0.890916146 |
| ko00281:Geraniol degradation | 153956173.4 | 103012312.8 | 0.914016582 |
| ko00590:Arachidonic acid metabolism | 229817.9841 | 5709.579013 | 0.941985728 |
| ko03015:mRNA surveillance pathway | 135926.7822 | 75264.671 | 0.944007964 |
| ko04113:Meiosis - yeast | 429687.6709 | 378554.2733 | 0.995968007 |
