# Supplementary material for: Identification of intestinal microbiome associated with lymph-vascular invasion in colorectal cancer patients and predictive label construction
Source: Front Cell Infect Microbiol. 2023 May 12;13:1098310. doi: 10.3389/fcimb.2023.1098310 (PMC10215531; doi:10.3389/fcimb.2023.1098310)
Supplement: Supplementary Table 5 — List of differential GO items of CRC patients stratified by LVI condition. [file Table_5.docx]

**Supplementary Table 5. List of differential KEGG pathways of CRC patients stratified by LVI condition**

| KEGG Pathways | logFC | P.Value |
| --- | --- | --- |
| KEGG_GALACTOSE_METABOLISM | 0.02049 | 0.025304 |
| KEGG_OTHER_GLYCAN_DEGRADATION | -0.02723 | 0.02606 |
| KEGG_ALDOSTERONE_REGULATED_SODIUM_REABSORPTION | 0.031345 | 0.027382 |
| KEGG_CYTOSOLIC_DNA_SENSING_PATHWAY | 0.042381 | 0.035677 |
