# Supplementary material for: Identification of intestinal microbiome associated with lymph-vascular invasion in colorectal cancer patients and predictive label construction
Source: Front Cell Infect Microbiol. 2023 May 12;13:1098310. doi: 10.3389/fcimb.2023.1098310 (PMC10215531; doi:10.3389/fcimb.2023.1098310)
Supplement: Supplementary Table 6 — List of differential KEGG pathways of CRC patients stratified by LVI condition. [file Table_6.docx]

**Supplementary Table 6. List of differential GO items of CRC patients stratified by LVI condition**

| GO items | LogFC | P.Value |
| --- | --- | --- |
| GOMF_FATTY_ACID_TRANSMEMBRANE_TRANSPORTER_ACTIVITY | 0.071956 | 6.82E-06 |
| GOMF_WATER_TRANSMEMBRANE_TRANSPORTER_ACTIVITY | 0.089172 | 0.000106 |
| GOBP_POLYOL_TRANSMEMBRANE_TRANSPORT | 0.060248 | 0.000199 |
| GOBP_POSITIVE_REGULATION_OF_LEUKOCYTE_DEGRANULATION | 0.047359 | 0.000263 |
| GOBP_NEUTROPHIL_DEGRANULATION | 0.06823 | 0.000333 |
| GOBP_NEUTROPHIL_ACTIVATION_INVOLVED_IN_IMMUNE_RESPONSE | 0.066582 | 0.0004 |
| GOBP_NEUTROPHIL_MEDIATED_IMMUNITY | 0.052557 | 0.000415 |
| GOBP_REGULATION_OF_FAT_CELL_PROLIFERATION | 0.062998 | 0.000466 |
| GOBP_POSITIVE_REGULATION_OF_GRANULOCYTE_CHEMOTAXIS | 0.059046 | 0.00065 |
| GOBP_REGULATION_OF_RESTING_MEMBRANE_POTENTIAL | 0.060106 | 0.000788 |
| GOBP_REGULATION_OF_DOPAMINERGIC_NEURON_DIFFERENTIATION | 0.098199 | 0.000804 |
| GOBP_DOPAMINERGIC_NEURON_DIFFERENTIATION | 0.041553 | 0.000817 |
| GOMF_WATER_CHANNEL_ACTIVITY | 0.093991 | 0.001158 |
| GOMF_POLYOL_TRANSMEMBRANE_TRANSPORTER_ACTIVITY | 0.046888 | 0.001397 |
| GOMF_INTERLEUKIN_1_BINDING | 0.074123 | 0.001501 |
| GOBP_REGULATION_OF_HETEROTYPIC_CELL_CELL_ADHESION | 0.039273 | 0.001515 |
| GOBP_NEGATIVE_REGULATION_OF_CHEMOKINE_PRODUCTION | 0.05128 | 0.001636 |
| GOBP_LABYRINTHINE_LAYER_MORPHOGENESIS | 0.038186 | 0.001636 |
| GOCC_CILIARY_TIP | -0.03321 | 0.001715 |
| GOBP_CELL_CELL_ADHESION_INVOLVED_IN_GASTRULATION | 0.049534 | 0.001827 |
| GOBP_UDP_N_ACETYLGLUCOSAMINE_METABOLIC_PROCESS | 0.046817 | 0.001873 |
| GOBP_GRANULOCYTE_ACTIVATION | 0.067857 | 0.001939 |
| GOBP_CELLULAR_HYPOTONIC_RESPONSE | 0.069241 | 0.002088 |
| GOBP_PSEUDOPODIUM_ORGANIZATION | 0.038646 | 0.002209 |
| GOBP_CELLULAR_RESPONSE_TO_GLUCAGON_STIMULUS | 0.035856 | 0.002261 |
| GOMF_CARBOHYDRATE_TRANSMEMBRANE_TRANSPORTER_ACTIVITY | 0.029995 | 0.002373 |
| GOBP_NEGATIVE_REGULATION_OF_STRESS_ACTIVATED_PROTEIN_KINASE_SIGNALING_CASCADE | 0.038402 | 0.002445 |
| GOMF_PROTEIN_TYROSINE_THREONINE_PHOSPHATASE_ACTIVITY | 0.079228 | 0.002455 |
| GOBP_KILLING_OF_CELLS_OF_ANOTHER_ORGANISM | 0.036668 | 0.002555 |
| GOBP_HYPOTONIC_RESPONSE | 0.065551 | 0.002796 |
| GOBP_INTRACILIARY_TRANSPORT | -0.03722 | 0.002892 |
| GOBP_EMBRYONIC_PLACENTA_MORPHOGENESIS | 0.033276 | 0.002956 |
| GOBP_CELLULAR_RESPONSE_TO_LEUCINE_STARVATION | -0.04124 | 0.003075 |
| GOBP_REGULATION_OF_RELAXATION_OF_MUSCLE | 0.070678 | 0.003107 |
| GOBP_REGULATION_OF_SUPEROXIDE_METABOLIC_PROCESS | 0.046891 | 0.003383 |
| GOBP_RESPONSE_TO_PROSTAGLANDIN_E | 0.058669 | 0.003503 |
| GOBP_CELLULAR_RESPONSE_TO_OSMOTIC_STRESS | 0.028871 | 0.003574 |
| GOBP_RESPONSE_TO_ALDOSTERONE | 0.04377 | 0.003672 |
| GOBP_INTRACILIARY_ANTEROGRADE_TRANSPORT | -0.05136 | 0.003712 |
| GOBP_REGULATION_OF_SUPEROXIDE_ANION_GENERATION | 0.04354 | 0.003779 |
| GOBP_HETEROTYPIC_CELL_CELL_ADHESION | 0.040558 | 0.00383 |
| GOMF_MAP_KINASE_TYROSINE_SERINE_THREONINE_PHOSPHATASE_ACTIVITY | 0.059638 | 0.004031 |
| GOCC_INTRACILIARY_TRANSPORT_PARTICLE | -0.03766 | 0.004193 |
| GOBP_REGULATION_OF_PLASMA_MEMBRANE_ORGANIZATION | 0.028697 | 0.004216 |
| GOBP_POSITIVE_REGULATION_OF_MYELOID_LEUKOCYTE_MEDIATED_IMMUNITY | 0.07237 | 0.004361 |
| GOBP_POSITIVE_REGULATION_OF_POTASSIUM_ION_TRANSMEMBRANE_TRANSPORTER_ACTIVITY | 0.036844 | 0.004555 |
| GOCC_AXONEMAL_DYNEIN_COMPLEX | -0.03137 | 0.004865 |
| GOBP_MAGNESIUM_ION_HOMEOSTASIS | 0.059766 | 0.004895 |
| GOBP_CELL_MIGRATION_INVOLVED_IN_GASTRULATION | -0.05878 | 0.004953 |
| GOBP_NEGATIVE_REGULATION_OF_CELL_FATE_COMMITMENT | 0.078321 | 0.004972 |
| GOBP_POSITIVE_REGULATION_OF_T_HELPER_CELL_DIFFERENTIATION | 0.04967 | 0.005216 |
| GOBP_ANTIMICROBIAL_HUMORAL_IMMUNE_RESPONSE_MEDIATED_BY_ANTIMICROBIAL_PEPTIDE | 0.041335 | 0.005225 |
| GOBP_REGULATION_OF_INTEGRIN_ACTIVATION | 0.039862 | 0.005618 |
| GOBP_NATURAL_KILLER_CELL_MEDIATED_IMMUNE_RESPONSE_TO_TUMOR_CELL | 0.05546 | 0.005996 |
| GOMF_CHEMOKINE_ACTIVITY | 0.09033 | 0.006235 |
| GOBP_POSITIVE_REGULATION_OF_PSEUDOPODIUM_ASSEMBLY | 0.034195 | 0.006386 |
| GOMF_ACTIVIN_RECEPTOR_BINDING | -0.04691 | 0.006392 |
| GOBP_NEGATIVE_REGULATION_OF_CYTOKINE_PRODUCTION_INVOLVED_IN_INFLAMMATORY_RESPONSE | 0.044291 | 0.006583 |
| GOBP_B_CELL_APOPTOTIC_PROCESS | 0.028696 | 0.006923 |
| GOBP_AMINO_SUGAR_BIOSYNTHETIC_PROCESS | 0.034447 | 0.007066 |
| GOBP_EOSINOPHIL_MIGRATION | 0.091089 | 0.007074 |
| GOBP_CELLULAR_RESPONSE_TO_STEROL | -0.04188 | 0.007196 |
| GOMF_MAP_KINASE_PHOSPHATASE_ACTIVITY | 0.051569 | 0.0072 |
| GOBP_BRANCHING_INVOLVED_IN_LABYRINTHINE_LAYER_MORPHOGENESIS | 0.046083 | 0.007209 |
| GOBP_OUTER_DYNEIN_ARM_ASSEMBLY | -0.02638 | 0.007806 |
| GOBP_REGULATION_OF_BIOLOGICAL_PROCESS_INVOLVED_IN_SYMBIOTIC_INTERACTION | 0.02451 | 0.007811 |
| GOMF_CHEMOKINE_RECEPTOR_BINDING | 0.070396 | 0.007951 |
| GOBP_RESPONSE_TO_OSMOTIC_STRESS | 0.023856 | 0.008089 |
| GOBP_NEGATIVE_REGULATION_OF_GLUCOSE_TRANSMEMBRANE_TRANSPORT | 0.043627 | 0.008141 |
| GOBP_MESODERMAL_CELL_FATE_SPECIFICATION | 0.052694 | 0.00815 |
| GOCC_INTRACILIARY_TRANSPORT_PARTICLE_B | -0.04017 | 0.008202 |
| GOBP_NUCLEOBASE_TRANSPORT | 0.057578 | 0.008293 |
| GOBP_POSITIVE_REGULATION_OF_STEROID_HORMONE_SECRETION | 0.088448 | 0.00836 |
| GOBP_POSITIVE_REGULATION_OF_T_CELL_DIFFERENTIATION_IN_THYMUS | 0.053553 | 0.008497 |
| GOBP_NEGATIVE_REGULATION_OF_JNK_CASCADE | 0.035918 | 0.008511 |
| GOBP_POSITIVE_REGULATION_OF_ENDOTHELIAL_CELL_DIFFERENTIATION | -0.04056 | 0.008662 |
| GOBP_NEGATIVE_REGULATION_OF_BONE_MINERALIZATION | 0.042607 | 0.00872 |
| GOBP_CELL_CELL_JUNCTION_MAINTENANCE | 0.057937 | 0.008733 |
| GOBP_REGULATION_OF_LEUKOCYTE_DEGRANULATION | 0.041289 | 0.008823 |
| GOBP_PURINE_NUCLEOBASE_METABOLIC_PROCESS | 0.031529 | 0.008925 |
| GOBP_UTERINE_SMOOTH_MUSCLE_CONTRACTION | -0.05777 | 0.008934 |
| GOBP_G_PROTEIN_COUPLED_GLUTAMATE_RECEPTOR_SIGNALING_PATHWAY | -0.04786 | 0.008983 |
| GOBP_MIDBRAIN_DOPAMINERGIC_NEURON_DIFFERENTIATION | 0.062045 | 0.009079 |
| GOBP_POSITIVE_REGULATION_OF_CELL_ADHESION_MEDIATED_BY_INTEGRIN | 0.060526 | 0.009334 |
| GOBP_ANTIMICROBIAL_HUMORAL_RESPONSE | 0.03166 | 0.009409 |
| GOCC_PHOTORECEPTOR_CONNECTING_CILIUM | -0.02817 | 0.009558 |
| GOBP_DEFENSE_RESPONSE_TO_GRAM_POSITIVE_BACTERIUM | 0.029139 | 0.00974 |
| GOBP_REGULATION_OF_NEUTROPHIL_ACTIVATION | 0.075157 | 0.009766 |
| GOBP_RESPONSE_TO_LEUCINE | -0.04092 | 0.010063 |
| GOBP_POSITIVE_REGULATION_OF_MIRNA_METABOLIC_PROCESS | 0.025568 | 0.010387 |
| GOBP_NEGATIVE_REGULATION_OF_MAPK_CASCADE | 0.025829 | 0.010496 |
| GOBP_MYELIN_MAINTENANCE | 0.034603 | 0.010613 |
| GOMF_PEPTIDYL_PROLINE_DIOXYGENASE_ACTIVITY | 0.033205 | 0.010844 |
| GOBP_REGULATION_OF_NK_T_CELL_ACTIVATION | 0.039945 | 0.01096 |
| GOBP_MYELOID_LEUKOCYTE_MEDIATED_IMMUNITY | 0.044065 | 0.010987 |
| GOMF_MYOSIN_V_BINDING | 0.031076 | 0.01099 |
| GOBP_NEGATIVE_REGULATION_OF_DIGESTIVE_SYSTEM_PROCESS | 0.044817 | 0.011145 |
| GOBP_PHOSPHATIDYLSERINE_METABOLIC_PROCESS | 0.029391 | 0.011183 |
| GOBP_REGULATION_OF_FEEDING_BEHAVIOR | 0.038793 | 0.011306 |
| GOBP_INTERCELLULAR_TRANSPORT | 0.041497 | 0.011318 |
| GOBP_NEGATIVE_REGULATION_OF_VIRAL_ENTRY_INTO_HOST_CELL | 0.028959 | 0.011397 |
| GOBP_CELLULAR_HYPEROSMOTIC_RESPONSE | 0.037177 | 0.011583 |
| GOBP_ACTIN_FILAMENT_SEVERING | 0.02922 | 0.011742 |
| GOMF_ARP2_3_COMPLEX_BINDING | 0.02894 | 0.01197 |
| GOCC_CILIARY_BASE | -0.02605 | 0.012236 |
| GOBP_REGULATION_OF_TOLL_LIKE_RECEPTOR_2_SIGNALING_PATHWAY | 0.047642 | 0.012237 |
| GOBP_FRUCTOSE_6_PHOSPHATE_METABOLIC_PROCESS | 0.051526 | 0.0123 |
| GOBP_DENTATE_GYRUS_DEVELOPMENT | -0.04624 | 0.012747 |
| GOBP_EOSINOPHIL_CHEMOTAXIS | 0.087629 | 0.012753 |
| GOBP_NEGATIVE_REGULATION_OF_ERK1_AND_ERK2_CASCADE | 0.028234 | 0.01336 |
| GOBP_NEGATIVE_REGULATION_OF_ALCOHOL_BIOSYNTHETIC_PROCESS | 0.033283 | 0.013533 |
| GOBP_POSITIVE_REGULATION_OF_NEUTROPHIL_MIGRATION | 0.045461 | 0.014183 |
| GOCC_CILIARY_TRANSITION_ZONE | -0.02777 | 0.014293 |
| GOBP_RESPONSE_TO_INTERLEUKIN_6 | 0.027062 | 0.014363 |
| GOBP_CELLULAR_RESPONSE_TO_KETONE | 0.028581 | 0.014602 |
| GOBP_FAT_CELL_PROLIFERATION | 0.043327 | 0.014857 |
| GOMF_NEUROTRANSMITTER_RECEPTOR_REGULATOR_ACTIVITY | -0.07377 | 0.014954 |
| GOBP_LEUKOCYTE_DEGRANULATION | 0.049962 | 0.015085 |
| GOBP_POSITIVE_REGULATION_OF_MIRNA_TRANSCRIPTION | 0.025993 | 0.015178 |
| GOBP_NEGATIVE_REGULATION_OF_GLUCOSE_IMPORT | 0.033854 | 0.015229 |
| GOBP_POSITIVE_REGULATION_OF_PHOSPHATASE_ACTIVITY | 0.02964 | 0.015231 |
| GOBP_REGULATION_OF_STEROID_HORMONE_SECRETION | 0.033427 | 0.015407 |
| GOBP_LYMPHOCYTE_CHEMOTAXIS | 0.071721 | 0.015721 |
| GOBP_CELLULAR_RESPONSE_TO_TESTOSTERONE_STIMULUS | 0.045513 | 0.01584 |
| GOMF_RETINAL_BINDING | -0.06537 | 0.015969 |
| GOBP_CELLULAR_RESPONSE_TO_PROSTAGLANDIN_E_STIMULUS | 0.042643 | 0.016058 |
| GOCC_DYNEIN_COMPLEX | -0.02217 | 0.016298 |
| GOBP_REGULATION_OF_PHOSPHATIDYLCHOLINE_METABOLIC_PROCESS | 0.040928 | 0.016667 |
| GOBP_WATER_TRANSPORT | 0.046639 | 0.016779 |
| GOCC_SPERM_CONNECTING_PIECE | -0.04672 | 0.016809 |
| GOBP_GRANULOCYTE_MACROPHAGE_COLONY_STIMULATING_FACTOR_PRODUCTION | 0.037409 | 0.016895 |
| GOBP_MESODERMAL_CELL_FATE_COMMITMENT | 0.042845 | 0.017049 |
| GOBP_NEGATIVE_REGULATION_OF_LIPID_LOCALIZATION | 0.021068 | 0.017087 |
| GOBP_CELLULAR_RESPONSE_TO_OXIDISED_LOW_DENSITY_LIPOPROTEIN_PARTICLE_STIMULUS | 0.03835 | 0.017128 |
| GOBP_CELLULAR_RESPONSE_TO_INTERLEUKIN_1 | 0.046462 | 0.017132 |
| GOMF_PROTON_CHANNEL_ACTIVITY | 0.046035 | 0.017231 |
| GOBP_NEGATIVE_REGULATION_OF_ALPHA_BETA_T_CELL_PROLIFERATION | 0.029122 | 0.017308 |
| GOBP_TOLL_LIKE_RECEPTOR_2_SIGNALING_PATHWAY | 0.050249 | 0.017386 |
| GOBP_CELLULAR_RESPONSE_TO_CORTICOSTEROID_STIMULUS | 0.026735 | 0.017712 |
| GOBP_ONE_CARBON_METABOLIC_PROCESS | 0.050336 | 0.017714 |
| GOBP_POSITIVE_REGULATION_OF_INSULIN_SECRETION_INVOLVED_IN_CELLULAR_RESPONSE_TO_GLUCOSE_STIMULUS | 0.039765 | 0.017848 |
| GOBP_RESPONSE_TO_PROSTAGLANDIN | 0.042595 | 0.018074 |
| GOBP_REGULATION_OF_POTASSIUM_ION_TRANSMEMBRANE_TRANSPORTER_ACTIVITY | 0.017631 | 0.0181 |
| GOBP_NEGATIVE_REGULATION_OF_VIRAL_LIFE_CYCLE | 0.028899 | 0.018463 |
| GOCC_TERMINAL_BOUTON | 0.024907 | 0.018658 |
| GOBP_REGULATION_OF_MEMBRANE_INVAGINATION | 0.062207 | 0.018764 |
| GOBP_NEGATIVE_REGULATION_OF_INTERLEUKIN_1_PRODUCTION | 0.036562 | 0.018947 |
| GOBP_REGULATION_OF_GLUCOCORTICOID_SECRETION | 0.043119 | 0.018961 |
| GOBP_MUCUS_SECRETION | 0.044125 | 0.019005 |
| GOMF_AMP_BINDING | 0.033029 | 0.019174 |
| GOBP_SMOOTHENED_SIGNALING_PATHWAY_INVOLVED_IN_DORSAL_VENTRAL_NEURAL_TUBE_PATTERNING | -0.03002 | 0.019361 |
| GOBP_NEGATIVE_REGULATION_OF_CD4_POSITIVE_ALPHA_BETA_T_CELL_PROLIFERATION | 0.035031 | 0.019364 |
| GOBP_REGULATION_OF_CALCIUM_ION_IMPORT | 0.034479 | 0.019642 |
| GOMF_SUGAR_TRANSMEMBRANE_TRANSPORTER_ACTIVITY | 0.029357 | 0.019904 |
| GOBP_INTRACILIARY_RETROGRADE_TRANSPORT | -0.03092 | 0.019962 |
| GOMF_CYTOKINE_ACTIVITY | 0.030121 | 0.020076 |
| GOBP_POSITIVE_REGULATION_OF_REACTIVE_OXYGEN_SPECIES_METABOLIC_PROCESS | 0.02227 | 0.020096 |
| GOBP_REGULATION_OF_B_CELL_APOPTOTIC_PROCESS | 0.030921 | 0.020346 |
| GOBP_OUTER_MITOCHONDRIAL_MEMBRANE_ORGANIZATION | 0.0363 | 0.020732 |
| GOMF_NEUROPEPTIDE_RECEPTOR_ACTIVITY | 0.037093 | 0.021011 |
| GOBP_CELLULAR_RESPONSE_TO_LIPOPROTEIN_PARTICLE_STIMULUS | 0.027645 | 0.021132 |
| GOBP_NUCLEOSIDE_MONOPHOSPHATE_CATABOLIC_PROCESS | 0.041803 | 0.021162 |
| GOBP_REGULATION_OF_TOLL_LIKE_RECEPTOR_4_SIGNALING_PATHWAY | 0.029396 | 0.021321 |
| GOBP_REGULATION_OF_PRESYNAPTIC_CYTOSOLIC_CALCIUM_ION_CONCENTRATION | 0.076266 | 0.021328 |
| GOBP_REGULATION_OF_LYMPHOCYTE_CHEMOTAXIS | 0.088005 | 0.021454 |
| GOBP_RIBONUCLEOSIDE_MONOPHOSPHATE_CATABOLIC_PROCESS | 0.052616 | 0.021541 |
| GOMF_DYNEIN_LIGHT_INTERMEDIATE_CHAIN_BINDING | -0.02483 | 0.021542 |
| GOBP_CELLULAR_RESPONSE_TO_INTERFERON_GAMMA | 0.047734 | 0.021659 |
| GOBP_POSITIVE_REGULATION_OF_LYMPHOCYTE_CHEMOTAXIS | 0.08001 | 0.0217 |
| GOBP_POSITIVE_REGULATION_OF_CD4_POSITIVE_ALPHA_BETA_T_CELL_DIFFERENTIATION | 0.045705 | 0.021705 |
| GOBP_POSITIVE_REGULATION_OF_HOMOTYPIC_CELL_CELL_ADHESION | 0.058484 | 0.021765 |
| GOBP_NEGATIVE_REGULATION_OF_MACROPHAGE_MIGRATION | 0.042206 | 0.021869 |
| GOMF_CCR_CHEMOKINE_RECEPTOR_BINDING | 0.075628 | 0.02211 |
| GOBP_CARBOHYDRATE_TRANSMEMBRANE_TRANSPORT | 0.021566 | 0.022114 |
| GOBP_THROMBIN_ACTIVATED_RECEPTOR_SIGNALING_PATHWAY | 0.046288 | 0.022471 |
| GOBP_SUPEROXIDE_ANION_GENERATION | 0.035817 | 0.022703 |
| GOBP_NEGATIVE_REGULATION_OF_JUN_KINASE_ACTIVITY | 0.056168 | 0.022887 |
| GOBP_MESODERMAL_CELL_DIFFERENTIATION | 0.025184 | 0.023039 |
| GOBP_PEPTIDYL_PROLINE_HYDROXYLATION | 0.024792 | 0.023111 |
| GOBP_RESPONSE_TO_CHEMOKINE | 0.063378 | 0.023879 |
| GOBP_REGULATION_OF_MYELOID_LEUKOCYTE_MEDIATED_IMMUNITY | 0.036758 | 0.02395 |
| GOBP_VERY_LOW_DENSITY_LIPOPROTEIN_PARTICLE_ASSEMBLY | 0.042891 | 0.023985 |
| GOBP_LONG_CHAIN_FATTY_ACID_IMPORT_ACROSS_PLASMA_MEMBRANE | 0.037686 | 0.024174 |
| GOMF_PEPTIDOGLYCAN_BINDING | 0.082568 | 0.024418 |
| GOBP_RESPONSE_TO_GRAVITY | 0.047357 | 0.024543 |
| GOMF_MOLECULAR_FUNCTION_ACTIVATOR_ACTIVITY | 0.022679 | 0.02473 |
| GOBP_RESPONSE_TO_ANGIOTENSIN | 0.028366 | 0.024888 |
| GOBP_SUPEROXIDE_METABOLIC_PROCESS | 0.026144 | 0.024916 |
| GOBP_RESPONSE_TO_TUMOR_NECROSIS_FACTOR | 0.027732 | 0.024963 |
| GOBP_REGULATION_OF_RESPONSE_TO_EXTRACELLULAR_STIMULUS | -0.02642 | 0.025065 |
| GOBP_DETECTION_OF_TEMPERATURE_STIMULUS_INVOLVED_IN_SENSORY_PERCEPTION_OF_PAIN | 0.059961 | 0.025206 |
| GOBP_NK_T_CELL_ACTIVATION | 0.029173 | 0.02523 |
| GOBP_NON_MOTILE_CILIUM_ASSEMBLY | -0.02402 | 0.025251 |
| GOBP_DETECTION_OF_STIMULUS_INVOLVED_IN_SENSORY_PERCEPTION_OF_PAIN | 0.042478 | 0.025527 |
| GOBP_DETECTION_OF_VISIBLE_LIGHT | -0.01691 | 0.025684 |
| GOBP_REGULATION_OF_POSITIVE_CHEMOTAXIS | 0.02527 | 0.025817 |
| GOBP_CORTICAL_ACTIN_CYTOSKELETON_ORGANIZATION | 0.025295 | 0.025859 |
| GOBP_PEROXISOME_PROLIFERATOR_ACTIVATED_RECEPTOR_SIGNALING_PATHWAY | 0.02108 | 0.025872 |
| GOBP_NEGATIVE_REGULATION_OF_CYSTEINE_TYPE_ENDOPEPTIDASE_ACTIVITY | 0.018078 | 0.0259 |
| GOBP_DNA_METHYLATION_INVOLVED_IN_GAMETE_GENERATION | -0.02113 | 0.026017 |
| GOBP_ENTRY_INTO_HOST | 0.02305 | 0.026053 |
| GOBP_POSITIVE_REGULATION_OF_PROTEIN_SECRETION | 0.018954 | 0.026122 |
| GOBP_CELLULAR_RESPONSE_TO_LOW_DENSITY_LIPOPROTEIN_PARTICLE_STIMULUS | 0.027299 | 0.02624 |
| GOBP_POSITIVE_REGULATION_OF_NEUROINFLAMMATORY_RESPONSE | 0.080076 | 0.026318 |
| GOMF_CASPASE_BINDING | 0.030843 | 0.026439 |
| GOBP_HOMOTYPIC_CELL_CELL_ADHESION | 0.0207 | 0.026548 |
| GOBP_REGULATION_OF_CARDIAC_MUSCLE_ADAPTATION | 0.038209 | 0.026652 |
| GOBP_RESPONSE_TO_INTERFERON_GAMMA | 0.047985 | 0.026665 |
| GOBP_POSITIVE_REGULATION_OF_CALCIUM_ION_IMPORT | 0.047137 | 0.026776 |
| GOBP_POSITIVE_REGULATION_OF_VIRAL_LIFE_CYCLE | 0.025104 | 0.026794 |
| GOMF_MINUS_END_DIRECTED_MICROTUBULE_MOTOR_ACTIVITY | -0.02325 | 0.026823 |
| GOBP_DETECTION_OF_TEMPERATURE_STIMULUS_INVOLVED_IN_SENSORY_PERCEPTION | 0.053541 | 0.026996 |
| GOBP_MYELOID_CELL_ACTIVATION_INVOLVED_IN_IMMUNE_RESPONSE | 0.041756 | 0.0271 |
| GOBP_RESPONSE_TO_MINERALOCORTICOID | 0.026157 | 0.027134 |
| GOBP_NATURAL_KILLER_CELL_CHEMOTAXIS | 0.141925 | 0.027236 |
| GOBP_NEUTROPHIL_MEDIATED_KILLING_OF_SYMBIONT_CELL | 0.064804 | 0.02736 |
| GOBP_POSITIVE_REGULATION_OF_GLYCOPROTEIN_METABOLIC_PROCESS | 0.03065 | 0.027365 |
| GOBP_POSITIVE_REGULATION_OF_NUCLEOTIDE_BIOSYNTHETIC_PROCESS | 0.048801 | 0.027471 |
| GOBP_POSITIVE_REGULATION_OF_CD4_POSITIVE_ALPHA_BETA_T_CELL_ACTIVATION | 0.04259 | 0.027569 |
| GOBP_REACTIVE_NITROGEN_SPECIES_METABOLIC_PROCESS | 0.027459 | 0.027616 |
| GOBP_REGULATION_OF_UNSATURATED_FATTY_ACID_BIOSYNTHETIC_PROCESS | 0.04714 | 0.027753 |
| GOBP_POSITIVE_REGULATION_OF_COLD_INDUCED_THERMOGENESIS | 0.016957 | 0.02785 |
| GOMF_CYCLASE_REGULATOR_ACTIVITY | 0.056328 | 0.02791 |
| GOBP_IMMUNOLOGICAL_MEMORY_PROCESS | 0.035033 | 0.027947 |
| GOBP_POSITIVE_REGULATION_OF_INTEGRIN_ACTIVATION | 0.043181 | 0.028387 |
| GOBP_NEGATIVE_REGULATION_OF_NERVOUS_SYSTEM_PROCESS | 0.027775 | 0.028578 |
| GOBP_NEGATIVE_REGULATION_OF_GTPASE_ACTIVITY | -0.021 | 0.028649 |
| GOBP_APICAL_PROTEIN_LOCALIZATION | -0.03241 | 0.028709 |
| GOBP_NEURONAL_ION_CHANNEL_CLUSTERING | -0.02269 | 0.0289 |
| GOBP_RESPONSE_TO_GLUCAGON | 0.035993 | 0.029224 |
| GOBP_SPECIFICATION_OF_SYMMETRY | -0.01989 | 0.029325 |
| GOBP_POSITIVE_REGULATION_OF_GUANYLATE_CYCLASE_ACTIVITY | 0.095465 | 0.029571 |
| GOMF_GLUCOSE_BINDING | 0.048174 | 0.029742 |
| GOBP_IMMUNOLOGICAL_MEMORY_FORMATION_PROCESS | 0.041844 | 0.029752 |
| GOBP_SINGLE_STRAND_BREAK_REPAIR | -0.02149 | 0.029824 |
| GOMF_L_ALANINE_TRANSMEMBRANE_TRANSPORTER_ACTIVITY | -0.02908 | 0.029966 |
| GOBP_NEPHRON_TUBULE_FORMATION | -0.0407 | 0.030058 |
| GOBP_VENTRICULAR_SYSTEM_DEVELOPMENT | -0.02082 | 0.030103 |
| GOBP_RESPONSE_TO_CADMIUM_ION | 0.034385 | 0.030309 |
| GOBP_RELAXATION_OF_MUSCLE | 0.038099 | 0.030316 |
| GOBP_NEGATIVE_REGULATION_OF_B_CELL_APOPTOTIC_PROCESS | 0.033887 | 0.030489 |
| GOBP_REGULATION_OF_PROTEIN_LOCALIZATION_TO_SYNAPSE | -0.02384 | 0.030705 |
| GOBP_REGULATION_OF_PROTEIN_EXPORT_FROM_NUCLEUS | 0.018881 | 0.030805 |
| GOBP_CELLULAR_RESPONSE_TO_PROSTAGLANDIN_STIMULUS | 0.032326 | 0.03108 |
| GOBP_DETECTION_OF_TEMPERATURE_STIMULUS | 0.047795 | 0.031134 |
| GOBP_POSITIVE_REGULATION_OF_PLATELET_AGGREGATION | 0.061164 | 0.031284 |
| GOCC_CHROMAFFIN_GRANULE | 0.055027 | 0.031414 |
| GOBP_INTERLEUKIN_6_MEDIATED_SIGNALING_PATHWAY | 0.030189 | 0.031499 |
| GOBP_REGULATION_OF_ACTIN_FILAMENT_BASED_MOVEMENT | 0.02287 | 0.031743 |
| GOBP_AMMONIUM_TRANSMEMBRANE_TRANSPORT | 0.029736 | 0.031745 |
| GOMF_AMMONIUM_TRANSMEMBRANE_TRANSPORTER_ACTIVITY | 0.029736 | 0.031745 |
| GOBP_PHAGOSOME_LYSOSOME_FUSION | -0.02296 | 0.031778 |
| GOBP_RHODOPSIN_MEDIATED_SIGNALING_PATHWAY | -0.01899 | 0.031814 |
| GOBP_CELLULAR_RESPONSE_TO_ALDEHYDE | 0.032162 | 0.031854 |
| GOBP_HYPEROSMOTIC_RESPONSE | 0.026962 | 0.031905 |
| GOBP_REGULATION_OF_T_CELL_DIFFERENTIATION_IN_THYMUS | 0.028417 | 0.032276 |
| GOBP_NEUTROPHIL_MIGRATION | 0.048854 | 0.032377 |
| GOBP_POSITIVE_REGULATION_OF_LIPID_TRANSPORT | 0.018341 | 0.03248 |
| GOMF_CALCIUM_ACTIVATED_CATION_CHANNEL_ACTIVITY | 0.017719 | 0.032703 |
| GOBP_RESPONSE_TO_INTERLEUKIN_1 | 0.037577 | 0.032811 |
| GOBP_REGULATION_OF_FC_RECEPTOR_MEDIATED_STIMULATORY_SIGNALING_PATHWAY | 0.057541 | 0.032908 |
| GOMF_OXIDOREDUCTASE_ACTIVITY_ACTING_ON_THE_CH_CH_GROUP_OF_DONORS_OXYGEN_AS_ACCEPTOR | -0.03082 | 0.032928 |
| GOBP_POSITIVE_REGULATION_OF_AMYLOID_PRECURSOR_PROTEIN_CATABOLIC_PROCESS | 0.030775 | 0.033033 |
| GOCC_INTRINSIC_COMPONENT_OF_EXTERNAL_SIDE_OF_PLASMA_MEMBRANE | 0.036267 | 0.033046 |
| GOBP_DETECTION_OF_LIGHT_STIMULUS | -0.01651 | 0.033135 |
| GOBP_MALE_SEX_DETERMINATION | 0.017392 | 0.033143 |
| GOBP_L_SERINE_METABOLIC_PROCESS | -0.04591 | 0.033185 |
| GOBP_NEGATIVE_REGULATION_OF_PEPTIDYL_TYROSINE_PHOSPHORYLATION | 0.022339 | 0.033383 |
| GOBP_SENSORY_PERCEPTION_OF_TEMPERATURE_STIMULUS | 0.035943 | 0.03364 |
| GOBP_NEGATIVE_REGULATION_OF_TOLL_LIKE_RECEPTOR_4_SIGNALING_PATHWAY | 0.045328 | 0.033699 |
| GOMF_CATECHOLAMINE_BINDING | -0.05118 | 0.033861 |
| GOBP_REGULATION_OF_VIRAL_ENTRY_INTO_HOST_CELL | 0.02144 | 0.033984 |
| GOCC_SPECIFIC_GRANULE | 0.025233 | 0.033992 |
| GOBP_POSITIVE_REGULATION_OF_TYROSINE_PHOSPHORYLATION_OF_STAT_PROTEIN | 0.023375 | 0.034025 |
| GOBP_PROTEIN_LOCALIZATION_TO_POSTSYNAPSE | -0.01926 | 0.03421 |
| GOBP_EPITHELIAL_CELL_PROLIFERATION_INVOLVED_IN_LUNG_MORPHOGENESIS | 0.022434 | 0.034412 |
| GOBP_KERATINOCYTE_MIGRATION | 0.03347 | 0.034458 |
| GOBP_NEGATIVE_REGULATION_OF_BEHAVIOR | 0.051521 | 0.034479 |
| GOBP_PHOSPHATIDYLCHOLINE_CATABOLIC_PROCESS | 0.035133 | 0.034863 |
| GOBP_PRO_B_CELL_DIFFERENTIATION | -0.02183 | 0.034901 |
| GOMF_CARBONATE_DEHYDRATASE_ACTIVITY | 0.087983 | 0.034914 |
| GOBP_NEGATIVE_REGULATION_OF_SMOOTH_MUSCLE_CONTRACTION | 0.042937 | 0.035096 |
| GOBP_ESTABLISHMENT_OF_LYMPHOCYTE_POLARITY | 0.051127 | 0.035099 |
| GOBP_CELLULAR_RESPONSE_TO_REACTIVE_OXYGEN_SPECIES | 0.015444 | 0.035479 |
| GOBP_EPIDERMIS_MORPHOGENESIS | 0.027624 | 0.035669 |
| GOBP_CELL_KILLING | 0.034035 | 0.035676 |
| GOBP_GRANULOCYTE_MIGRATION | 0.046248 | 0.035861 |
| GOBP_T_CELL_CHEMOTAXIS | 0.073755 | 0.035869 |
| GOBP_CELLULAR_RESPONSE_TO_MINERALOCORTICOID_STIMULUS | 0.034483 | 0.035985 |
| GOCC_AP_1_ADAPTOR_COMPLEX | 0.032542 | 0.036115 |
| GOCC_CELL_BODY_MEMBRANE | 0.021049 | 0.036618 |
| GOBP_PHOSPHATIDYLCHOLINE_METABOLIC_PROCESS | 0.025158 | 0.036699 |
| GOBP_CYTOKINE_PRODUCTION_INVOLVED_IN_INFLAMMATORY_RESPONSE | 0.037128 | 0.036757 |
| GOBP_POSITIVE_REGULATION_OF_ERBB_SIGNALING_PATHWAY | 0.018772 | 0.037147 |
| GOMF_ABC_TYPE_XENOBIOTIC_TRANSPORTER_ACTIVITY | -0.02821 | 0.037426 |
| GOBP_ACTIN_CROSSLINK_FORMATION | 0.032629 | 0.037464 |
| GOBP_VESTIBULOCOCHLEAR_NERVE_DEVELOPMENT | 0.035683 | 0.037617 |
| GOBP_CELLULAR_RESPONSE_TO_HYDROGEN_PEROXIDE | 0.016865 | 0.0377 |
| GOBP_PURINE_NUCLEOSIDE_MONOPHOSPHATE_CATABOLIC_PROCESS | 0.047733 | 0.037933 |
| GOBP_REGULATION_OF_RESPONSE_TO_TUMOR_CELL | 0.039928 | 0.037977 |
| GOBP_CELL_DIFFERENTIATION_INVOLVED_IN_EMBRYONIC_PLACENTA_DEVELOPMENT | 0.03002 | 0.038097 |
| GOMF_PROTEASE_BINDING | 0.018706 | 0.03824 |
| GOBP_POSITIVE_REGULATION_OF_NUCLEOTIDE_METABOLIC_PROCESS | 0.027134 | 0.038295 |
| GOBP_DETECTION_OF_LIGHT_STIMULUS_INVOLVED_IN_SENSORY_PERCEPTION | -0.03068 | 0.038319 |
| GOBP_SYNAPTIC_TRANSMISSION_GABAERGIC | 0.01762 | 0.038344 |
| GOBP_REGULATION_OF_PLASMA_LIPOPROTEIN_PARTICLE_LEVELS | 0.018448 | 0.038779 |
| GOBP_CELLULAR_RESPONSE_TO_DEXAMETHASONE_STIMULUS | 0.031158 | 0.03879 |
| GOMF_DYNEIN_HEAVY_CHAIN_BINDING | -0.01634 | 0.038868 |
| GOBP_COBALAMIN_METABOLIC_PROCESS | -0.02396 | 0.038953 |
| GOBP_LEUKOCYTE_CHEMOTAXIS | 0.040185 | 0.039227 |
| GOMF_LOW_DENSITY_LIPOPROTEIN_PARTICLE_BINDING | 0.049745 | 0.03931 |
| GOBP_CARBOHYDRATE_TRANSPORT | 0.024791 | 0.039315 |
| GOBP_SECONDARY_HEART_FIELD_SPECIFICATION | -0.04801 | 0.039357 |
| GOBP_POSITIVE_REGULATION_OF_GRANULOCYTE_MACROPHAGE_COLONY_STIMULATING_FACTOR_PRODUCTION | 0.034589 | 0.039551 |
| GOBP_PHENOL_CONTAINING_COMPOUND_CATABOLIC_PROCESS | -0.04028 | 0.039599 |
| GOBP_RESPONSE_TO_ORGANOPHOSPHORUS | 0.016871 | 0.039918 |
| GOBP_NADH_REGENERATION | 0.027991 | 0.03997 |
| GOBP_POSITIVE_REGULATION_OF_BEHAVIOR | -0.02802 | 0.040133 |
| GOBP_POSITIVE_REGULATION_OF_NITRIC_OXIDE_METABOLIC_PROCESS | 0.029265 | 0.040165 |
| GOMF_EXOGENOUS_PROTEIN_BINDING | 0.022899 | 0.040455 |
| GOBP_RESPONSE_TO_INTERLEUKIN_17 | 0.031688 | 0.040727 |
| GOMF_METHYLATED_HISTONE_BINDING | -0.02378 | 0.040813 |
| GOMF_NEUROTROPHIN_BINDING | 0.032827 | 0.040882 |
| GOBP_ERK1_AND_ERK2_CASCADE | 0.02352 | 0.040988 |
| GOBP_DEOXYRIBONUCLEOSIDE_MONOPHOSPHATE_CATABOLIC_PROCESS | 0.038865 | 0.04109 |
| GOCC_9PLUS0_NON_MOTILE_CILIUM | -0.01679 | 0.041412 |
| GOBP_NEUTROPHIL_CHEMOTAXIS | 0.052386 | 0.041694 |
| GOBP_DEFENSE_RESPONSE_TO_FUNGUS | 0.023143 | 0.041946 |
| GOBP_POSITIVE_REGULATION_OF_RIG_I_SIGNALING_PATHWAY | 0.03165 | 0.042211 |
| GOBP_NEGATIVE_REGULATION_BY_HOST_OF_VIRAL_TRANSCRIPTION | 0.044678 | 0.042317 |
| GOBP_REGULATION_OF_ERBB_SIGNALING_PATHWAY | 0.021603 | 0.042335 |
| GOBP_MOTOR_BEHAVIOR | 0.029156 | 0.042364 |
| GOBP_NEGATIVE_REGULATION_OF_MUSCLE_CONTRACTION | 0.032718 | 0.042405 |
| GOBP_POSITIVE_REGULATION_OF_BONE_RESORPTION | 0.022691 | 0.042601 |
| GOBP_POSITIVE_REGULATION_OF_LEUKOCYTE_CHEMOTAXIS | 0.041965 | 0.04262 |
| GOBP_RESPONSE_TO_CORTICOSTEROID | 0.020289 | 0.042679 |
| GOBP_FLUID_TRANSPORT | 0.042234 | 0.042686 |
| GOMF_BASIC_AMINO_ACID_TRANSMEMBRANE_TRANSPORTER_ACTIVITY | -0.03391 | 0.042856 |
| GOBP_REGULATION_OF_NEUTROPHIL_CHEMOTAXIS | 0.047876 | 0.042957 |
| GOBP_DECIDUALIZATION | 0.029859 | 0.043295 |
| GOBP_DEFENSE_RESPONSE_TO_GRAM_NEGATIVE_BACTERIUM | 0.022795 | 0.04346 |
| GOMF_PROTEIN_PHOSPHATASE_2A_BINDING | 0.017914 | 0.043518 |
| GOBP_RESPIRATORY_BURST | 0.04459 | 0.043706 |
| GOBP_DORSAL_SPINAL_CORD_DEVELOPMENT | -0.03694 | 0.04384 |
| GOBP_NEGATIVE_REGULATION_OF_LEUKOCYTE_CHEMOTAXIS | 0.058541 | 0.043981 |
| GOBP_REGULATION_OF_HOMOTYPIC_CELL_CELL_ADHESION | 0.029504 | 0.044012 |
| GOBP_REGULATION_OF_MIRNA_METABOLIC_PROCESS | 0.017094 | 0.044253 |
| GOBP_REGULATION_OF_HISTONE_H3_K9_TRIMETHYLATION | -0.01558 | 0.044317 |
| GOBP_POSITIVE_REGULATION_OF_UBIQUITIN_PROTEIN_LIGASE_ACTIVITY | 0.034843 | 0.044476 |
| GOBP_DENDRITIC_SPINE_MAINTENANCE | 0.02206 | 0.044815 |
| GOBP_NEGATIVE_REGULATION_OF_ANOIKIS | 0.023161 | 0.044867 |
| GOBP_POSITIVE_REGULATION_OF_NATURAL_KILLER_CELL_PROLIFERATION | 0.044118 | 0.044988 |
| GOBP_POLYOL_BIOSYNTHETIC_PROCESS | 0.01947 | 0.04527 |
| GOBP_POSITIVE_REGULATION_OF_VOLTAGE_GATED_POTASSIUM_CHANNEL_ACTIVITY | 0.033266 | 0.04536 |
| GOCC_SECRETORY_GRANULE | 0.019886 | 0.045416 |
| GOBP_UTERUS_DEVELOPMENT | -0.0422 | 0.045601 |
| GOBP_NEGATIVE_REGULATION_OF_HORMONE_SECRETION | 0.024247 | 0.045747 |
| GOCC_SERINE_TYPE_PEPTIDASE_COMPLEX | 0.042372 | 0.045799 |
| GOBP_RESPONSE_TO_UV_B | -0.02199 | 0.045888 |
| GOBP_REGULATION_OF_GRANULOCYTE_CHEMOTAXIS | 0.043307 | 0.046027 |
| GOBP_NEGATIVE_REGULATION_OF_STEROL_TRANSPORT | 0.035183 | 0.046442 |
| GOBP_GENITALIA_DEVELOPMENT | -0.02225 | 0.046618 |
| GOBP_PROTEIN_KINASE_C_ACTIVATING_G_PROTEIN_COUPLED_RECEPTOR_SIGNALING_PATHWAY | 0.016674 | 0.046669 |
| GOBP_LONG_CHAIN_FATTY_ACID_TRANSPORT | 0.024663 | 0.046785 |
| GOCC_TERTIARY_GRANULE | 0.028826 | 0.046838 |
| GOBP_T_CELL_ACTIVATION_INVOLVED_IN_IMMUNE_RESPONSE | 0.035863 | 0.047047 |
| GOBP_DEFENSE_RESPONSE_TO_BACTERIUM | 0.02798 | 0.047105 |
| GOBP_NEGATIVE_REGULATION_OF_RESPONSE_TO_CYTOKINE_STIMULUS | 0.032679 | 0.047112 |
| GOBP_NETRIN_ACTIVATED_SIGNALING_PATHWAY | -0.03612 | 0.047157 |
| GOBP_NEGATIVE_REGULATION_OF_CHOLESTEROL_STORAGE | 0.023574 | 0.047169 |
| GOBP_NEGATIVE_REGULATION_OF_MUSCLE_ADAPTATION | 0.032524 | 0.04721 |
| GOBP_POSITIVE_REGULATION_OF_CALCIUM_MEDIATED_SIGNALING | 0.035426 | 0.047215 |
| GOBP_POSITIVE_REGULATION_OF_ACTIVATED_T_CELL_PROLIFERATION | 0.019156 | 0.047309 |
| GOBP_SYNAPTONEMAL_COMPLEX_ORGANIZATION | -0.01873 | 0.047384 |
| GOBP_FATTY_ACID_TRANSMEMBRANE_TRANSPORT | 0.022911 | 0.047415 |
| GOBP_L_GLUTAMATE_IMPORT_ACROSS_PLASMA_MEMBRANE | 0.033346 | 0.048088 |
| GOBP_PURINE_CONTAINING_COMPOUND_CATABOLIC_PROCESS | 0.018151 | 0.048385 |
| GOBP_NEGATIVE_REGULATION_OF_LYMPHOCYTE_MIGRATION | 0.053159 | 0.048454 |
| GOBP_REGULATION_OF_LEUKOCYTE_CHEMOTAXIS | 0.042606 | 0.048502 |
| GOBP_POSITIVE_REGULATION_OF_FAT_CELL_DIFFERENTIATION | 0.021941 | 0.048614 |
| GOCC_TERTIARY_GRANULE_MEMBRANE | 0.047559 | 0.048699 |
| GOBP_MAINTENANCE_OF_BLOOD_BRAIN_BARRIER | 0.026073 | 0.048751 |
| GOBP_POSITIVE_REGULATION_OF_SYNAPTIC_TRANSMISSION_GABAERGIC | 0.033984 | 0.048812 |
| GOBP_MIRNA_METABOLIC_PROCESS | 0.015675 | 0.048895 |
| GOMF_PROTEIN_KINASE_B_BINDING | 0.021936 | 0.048911 |
| GOBP_POSITIVE_REGULATION_OF_WOUND_HEALING | 0.019855 | 0.049372 |
| GOBP_LABYRINTHINE_LAYER_DEVELOPMENT | 0.022302 | 0.049479 |
| GOBP_INTRINSIC_APOPTOTIC_SIGNALING_PATHWAY_IN_RESPONSE_TO_OXIDATIVE_STRESS | 0.021177 | 0.049494 |
| GOBP_MONOCYTE_CHEMOTAXIS | 0.067349 | 0.049658 |
| GOBP_CELLULAR_RESPONSE_TO_MACROPHAGE_COLONY_STIMULATING_FACTOR_STIMULUS | 0.063005 | 0.049673 |
| GOBP_GRANULOCYTE_CHEMOTAXIS | 0.048118 | 0.049727 |
| GOBP_INSULIN_METABOLIC_PROCESS | 0.037839 | 0.049964 |
